# Exploring Spatial and Temporal Variations in Stem-Mediated Greenhouse Gas Emissions from Different Species of Mangroves

**DOI:** 10.1021/acs.est.5c11290

**Published:** 2025-12-03

**Authors:** Zhao-Jun Yong, Wei-Jen Lin, Tzu-Chieh Chiu, Chen-Ying Ko, Pei-Luen Lu, Krisanadej Jaroensutasinee, Mullica Jaroensutasinee, Chiao-Wen Lin, Hsing-Juh Lin

**Affiliations:** 1 Department of Life Sciences and Innovation and Development Center of Sustainable Agriculture, 34916 National Chung Hsing University, Taichung 402202, Taiwan; 2 Department of Biological Resources, National Chiayi University, Chiayi 600355, Taiwan; 3 Department of Marine Environment and Engineering, 34874 National Sun Yat-sen University, Kaohsiung 804201, Taiwan; 4 Department of Life Science, 63285 National Taitung University, Taitung 950309, Taiwan; 5 Center of Excellence for Ecoinformatics, School of Science, 722944 Walailak University, Nakhon Si Thammarat 80160, Thailand

**Keywords:** mangrove forests, stem-mediated GHG fluxes, methane, nitrous oxide, carbon sink

## Abstract

Greenhouse gas (GHG) emissions, particularly methane (CH_4_) and nitrous oxide (N_2_O), from mangroves can challenge their carbon sequestration capacity. This study measured GHG emissions from the tree stems of four mangrove species (*Avicennia marina*, *Kandelia obovata*, *Lumnitzera racemosa*, and *Rhizophora stylosa*) along the western coast of Taiwan. All studied species emitted CO_2_, CH_4_, and N_2_O at the stem–atmosphere interface, with *A. marina* exhibiting the highest GHG fluxes. Notably, stem N_2_O emissions from *A. marina* significantly contributed (1.2 to 100.1%) to total ecosystem fluxes, whereas stem CH_4_ emissions were relatively low (−3.8 to 2.6%). This study is the first to demonstrate seasonal variations in stem N_2_O flux in mangroves, mainly influenced by temperature, as shown by the structural equation model. The results indicate that stem CH_4_ and N_2_O emissions can significantly offset soil carbon burial rates (0.19 to 889.28%), particularly in *A. marina*. First-order estimates suggest global mangrove stem-mediated emissions of 5.94 [0.88–168.65] Mg CH_4_ year^–1^ and 8.66 [2.46–36.19] Mg N_2_O year^–1^, presented as median [Q1–Q3] values, although these estimates carry some uncertainties. Nonetheless, these insights underscore the importance of including stem emissions in carbon budget assessments for mangrove ecosystems, highlighting their essential role in mitigating climate change.

## Graphical abstract

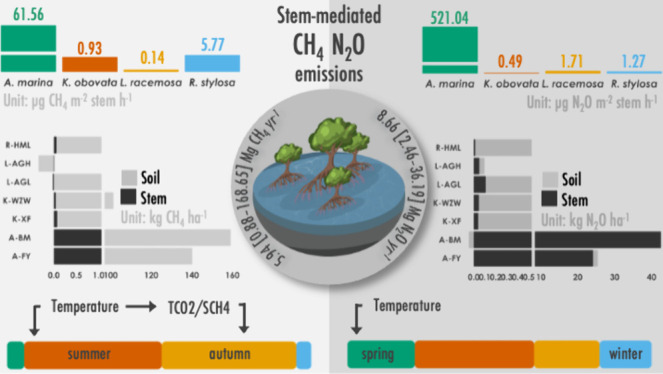

## Introduction

The concentrations of greenhouse gases (GHGs), which are the primary drivers of our current environmental crisis, have risen at an unprecedented rate.^1^ Notably, compared to carbon dioxide (CO_2_), methane (CH_4_) and nitrous oxide (N_2_O) have gained significant attention due to their much higher global warming potential (GWP), meaning that these gases are far more effective at trapping the Earth’s emitted thermal infrared radiation.^2^ The impact is even greater when considering sustained global warming potential (SGWP).^3^ In 2022, the average surface dry air mole fractions of atmospheric CO_2_, CH_4_, and N_2_O were approximately 1.50, 2.73, and 1.24 times greater than those seen in the preindustrial era, respectively.^4–6^ This increase has intensified the energy imbalance in our atmosphere,^7,8^ leading to a significant increase in global surface temperatures.^9,10^ Despite the goals set by the Paris Agreement to limit global temperature increases to less than 1.5 °C,^11^ the Intergovernmental Panel on Climate Change (IPCC) has confirmed that the ongoing escalation of global warming has led to irreversible changes.^2^ Therefore, substantial and sustained reductions in GHG emissions are essential for mitigating global warming.

Forests play a crucial role as carbon sinks by absorbing significant amounts of CO_2_ from the atmosphere through photosynthesis.^12,13^ While the carbon stored in biomass helps mitigate global warming, increased photosynthesis often leads to higher respiration rates,^14^ which can offset up to 82% of the absorbed carbon.^15^ Mangrove forests, characterized by woody plants that grow in the intertidal zones of tropical and subtropical regions, along with seagrass beds and salt marshes, are globally important vegetated coastal ecosystems (VCEs).^16,17^ The decomposition of organic matter occurs slowly because of waterlogged conditions,^18,19^ allowing VCEs to sequester “blue carbon” in the soil over longer time scales.^20^ Among VCEs, mangroves are particularly efficient at sequestering carbon.^21–23^ The carbon stock of mangroves is greater than that of tropical forests and exceeds the carbon stock of seagrass beds and salt marshes.^24–26^ However, the carbon stored in the soil is simultaneously released back into the atmosphere through heterotrophic respiration, which complicates the net fluxes between mangroves and the atmosphere.^21–23,27^

The anoxic environment of mangrove soils leads to contradictory outcomes: It helps preserve carbon while also making mangroves significant sources of CH_4_ and, to a lesser extent, N_2_O.^28–31^ Under these conditions, methanogenic archaea thrive, producing CH_4_ as a byproduct during methanogenesis, even as they compete with sulfate-reducing bacteria.^32,33^ Consequently, CH_4_ emissions from mangrove soils can reduce the carbon sink capacity of these forests due to their warming effect.^34–36^ Additionally, processes such as denitrification, nitrification, and nitrifier denitrification may contribute to N_2_O production.^37^ Mangroves can also be a source of N_2_O,^34,38–41^ with high nitrogen inputs potentially leading to greater N_2_O emissions than CH_4_.^29,42^

The main transport pathways for CH_4_ and N_2_O produced in deep soils include molecular diffusion through the water column, bubble ebullition, and aquatic plant transportation.^43,44^ Furthermore, the biological surfaces of trees can both emit and absorb CH_4_ and N_2_O, complicating their role in GHG dynamics within vegetated systems.^45,46^ Significant discrepancies remain between the top-down and bottom-up approaches used to estimate the global GHG budget, indicating a knowledge gap.^47,48^ The inclusion of tree-mediated CH_4_ fluxes may help resolve these discrepancies, highlighting the importance of this previously overlooked pathway.^49,50^ Interest in understanding the magnitudes, mechanisms, and sources of CH_4_ emitted from woody stem surfaces has increased since the 2000s,^49,51,52^ although research on stem-mediated N_2_O fluxes has received relatively little attention. CH_4_ and N_2_O emissions from tree stems can originate from both belowground and within the trees themselves.^53–56^ The mechanisms for axial transport of these gases include molecular diffusion, transpiration flow, and pressurized flow.^44,53,57^ Seasonal variations in stem CH_4_ fluxes can be influenced by temperature and vegetation dynamics.^58,59^ However, studies that measure stem GHG emissions over extended periods remain scarce. Moreover, despite the significant impact of stem-mediated CH_4_ emissions on ecosystems and the global CH_4_ budget, the contribution of this pathway remains unresolved.^60^ The challenges associated with measuring stem GHG fluxes in mangrove forests, compared with those in upland forests, have also limited our understanding of this pathway.^61–67^ Addressing these gaps is essential for accurately assessing the role of mangroves in blue carbon sinks and their broader implications for mitigating climate change.

The mangroves in Taiwan are distributed along the west coast, covering a total area of 939 ha.^25^ The mangrove species include *Avicennia marina* (Forsk.) Vierh, *Kandelia obovata* Sheue, Liu & Yong sp. nov, *Rhizophora stylosa* Griff, and *Lumnitzera racemosa* Willd.^68,69^ The objectives of this study are to (i) quantify GHG emissions from the stems of these four mangrove species with different root structures, (ii) explore the temporal and spatial variations in GHG emissions and influencing factors, (iii) clarify the sources and transport mechanisms of GHG emissions, and (iv) assess the impact of this pathway on the carbon fluxes of mangroves. We hypothesize that GHG emissions from the stems of mangrove species vary with the season and root structure.

## Methods and Materials

### Site Description

This study was conducted in mangroves distributed at seven sites along the western coast of Taiwan (Table 1 and Figure 1). K-WZW and K-XF are located in northern Taiwan, where *K. obovata* is the dominant species. The remaining sites are in southern Taiwan, with *A. marina* dominating A-FY and A-BM, *L. racemosa* in L-AGL and L-AGH, and *R. stylosa* in R-HML. Among the studied species, *A. marina* is the only mangrove species that has developed cone-like pneumatophores.^70^ However, *L. racemosa* in L-AGL and *R. stylosa* in R-HML have developed stilt roots.^71,72^

**Figure 1:**
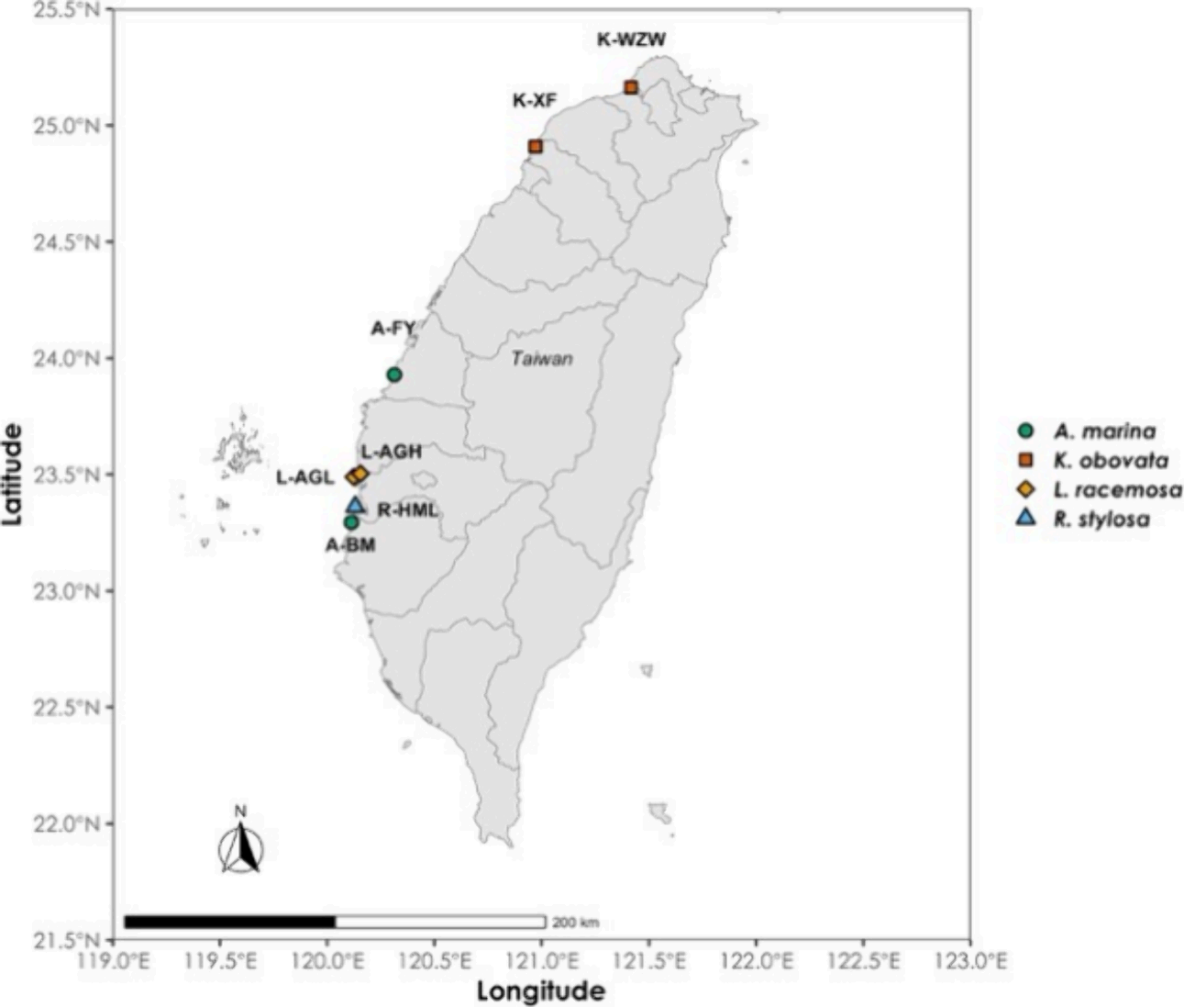
Sample sites along the western coast of Taiwan. Each species is represented by a distinct shape and color: green circles for *Avicennia marina*, red squares for *Kandelia obovata*, yellow diamonds for *Lumnitzera racemosa*, and a blue triangle for *Rhizophora stylosa*. The map was sourced from GADM (Global Administrative Areas, https://gadm.org), used under their academic use license.

**Table 1:** Information on the mangroves at the studied sites on the western coast of Taiwan

site		K-WZW	K-XF	A-FY	A-BM	L-AGL	L-AGH	R-HML
geographic coordinates		N25.16337°, E121.41689°	N24.90853°, E120.97193°	High: N23.92858°, E120.31381° Low: N23.92874°, E120.31303°	N23.29409°, E120.11276	N23.48939°, E120.12428°	N23.50361°, E120.15511°	N23.36111°, E120.13056°
sampling period		Jul 2021 to Sep 2022	Jul 2021 to Sep 2022	Apr 2021 to Oct 2022	May 2021 to Oct 2022	Apr 2023 to Apr 2024	Apr 2023 to Apr 2024	Jul 2023 to Dec 2024
mean seasonal rainfall (mm)^a^	Dec to Feb (winter)	163.5	101.0	41.2	44.5	10.7	10.7	10.0
	Mar to May (spring)	242.0	270.0	117.1	142.9	79.6	79.6	86.2
	Jun to Aug (summer)	153.5	182.6	225.8	311.3	138.2	138.2	220.6
	Sep to Nov (autumn)	196.9	57.5	12.8	17.1	61.5	61.5	54.0
mean seasonal temperature (°C)^a^	Dec to Feb (winter)	16.47	15.50	17.50	17.53	18.67	18.67	18.50
	Mar to May (spring)	21.30	20.40	23.88	24.45	24.20	24.20	24.33
	Jun to Aug (summer)	28.94	27.86	29.00	28.85	29.27	29.27	29.04
	Sep to Nov (autumn)	25.25	24.68	26.22	26.28	25.80	25.80	25.83
mean tidal range (cm)^a^		242	127	146	271	348	348	344
dominant mangrove species		*Kandelia obovota*	*Kandelia obovota*	*Avicennia marina*	*Avicennia marina*	*Lumnitzera racemosa*	*Lumnitzera racemosa*	*Rhizophora stylosa*
specialized root structure		none	none	pneumatophore	pneumatophore	stilt root	none	stilt root
mangrove forest area (ha)^b^		9.9	8.3	30.6	5.5	18.7	44.0	9.6
forest history (year)^b^		40	227	40	100	50	14	120
mean tree height (m)^b^		4.0	5.1	1.8	3.2	2.4	3.3	2.7
mean tree density (trees m^–2^)^b^		1.3	2.4	1.0	0.6	4.3	2.0	1.9
mean diameter at breast height (cm)^b^		7.0	5.6	10.5	6.2	2.6	3.4	2.4

a The data were sourced from the Central Weather Bureau of Taiwan. When the study period spanned more than 1 year, the seasonal rainfall and temperature were averaged. The tidal range reflects the average value from 2005 to 2024.

b The data were sourced from previous studies and unpublished data.^23,31^

L-AGH has the largest forest area (44.0 ha), while A-BM has the smallest forest area (5.5 ha). The mean tree height across all sites ranged from 1.8 to 5.1 m, and the average tree density and diameter at breast height (DBH) ranged from 0.6 to 2.4 trees m^–2^ and from 2.4 to 10.5 cm, respectively (Table 1). The tides were semidiurnal at all sites. However, L-AGL and L-AGH are not affected by the tidal cycle. Specifically, the mangroves at L-AGL are isolated from the outer sea by embankments, which are submerged only during the rainy season. L-AGH remained unsubmerged throughout the sampling campaign, and even a rainfall did not cause flooding.

### Flux Measurements

Sampling campaigns were conducted once each season, and data were collected over five seasons at each site (Table 1). Five to six mangrove trees were randomly selected within forests at each site for measuring the stem GHG fluxes of CO_2_, CH_4_, and N_2_O, considering site accessibility to minimize disturbances during data collection. At A-FY, three trees were located in lower elevation areas, while the other three were in higher elevation areas. Three sampling plots measuring 2 × 5 m and three plots measuring 5 × 5 m were established at L-AGL and L-AGH, respectively. The distance between the plots was at least 5 m. Two trees were selected from each sampling plot for measurements, except in one plot at L-AGL, where only one tree was chosen because of the low tree density. Similarly, three plots measuring 5 × 5 m were established at R-HML, with two trees selected from each plot. At A-BM, K-XF, and K-WZW, five trees were chosen at the sampling sites for flux measurement. These selected trees reflected the site’s general features, as they fell within the typical ranges of tree diameter and height. Moreover, by maintaining adequate distances between the selected trees, we ensured that our sampling captured a diverse range of conditions throughout the entire mangrove area, thereby enhancing the robustness and representativeness of our measurements.

In the mangrove forests of *K. obovata* and *A. marina*, measurements were conducted at height increments of 0–40, 40–80, 80–120, and 120–200 cm above the ground. However, owing to the height limitations of the trees, we established different height intervals for *L. racemosa* and *R. stylosa*. In *L. racemosa* mangrove forests, the height intervals were 0–30, 30–60, 60–90, and 90–120 cm above the ground. For *R. stylosa*, the height intervals were 0–40 and 40–80 cm above the ground. Due to differences in stem morphology, two distinct stem chambers were used to measure the GHG flux. A cylindrical chamber was used for *A. marina*, while a semirigid chamber, modified from the design by Siegenthaler et al.,^73^ was used for the other three species (Figure S1).

Two to three locations within 2 m of the sampled tree were selected to measure the soil GHG fluxes using the static chamber method.^74^ The soil chamber comprised a semicircular, transparent poly­(methyl methacrylate) (PMMA) cover (30 cm in diameter and 6.4 L in volume) and a stainless-steel ring (16 cm in height and 30 cm in diameter), with an adapter on the cover for connecting the tubing. The ring was pressed into the soil before the cover was placed over it, and long-tailed clips was used to secure and cover the steel ring tightly to achieve an airtight seal. At L-AGL, the water level was not influenced by the tide. Therefore, if the soil surface is inundated because of strong precipitation and if the water level exceeds 15 cm above ground, then the GHG flux across the water–atmosphere interface can be measured using the floating chamber method.^66,75^ The floating chamber consists of a cover identical to that of the soil chamber but is attached to a floating base made of ethylene-vinyl acetate (EVA) foam, which is 2 cm high. A detailed description of the floating chamber can be found in Yong et al.^66^

In this study, a portable gas analyzer (LI-7810; LI-COR Biosciences, Lincoln, Nebraska, USA) was used to simultaneously measure the CO_2_ and CH_4_ fluxes. N_2_O fluxes were measured by using N_2_O/H_2_O trace gas analyzers (LI-7820; LI-COR Bioscience, Nebraska, USA). The chamber was connected to the analyzer through tubing, and the gas inside the chamber was drawn into the analyzer with a pump, with each measurement lasting approximately 5 min to minimize the heating effects within the chamber. During each measurement, we counted the number of lenticels on the bark within the stem chamber in the field using a magnifying glass. We also visually counted the number of pneumatophores within the soil chamber of *A. marina*. After each measurement was completed, the airtight sealed chamber was opened to allow the GHG concentration within the chamber to stabilize. Precautions were taken to avoid exerting downward pressure or approaching the chamber closely, thereby preventing any unintended increase in GHG efflux. Sampling was conducted mainly during daylight hours and low tides. The GHG flux (F; mg m^–2^ h^–1^) was calculated using eq 1:

$$F=\frac{\left(S\times V\times c\right)}{R\times T\times A}$$ 1

where *S* is the slope obtained from the linear regression of GHG concentration changes over time (expressed in ppb CH_4_/N_2_O s^–1^ or ppm of CO_2_ s^–1^), *V* is the chamber volume (liters), *c* is the conversion factor from seconds to hours, *R* is the ideal gas constant (0.082 L atm K^–1^ mol^–1^), *T* is the air temperature inside the chamber (kelvin), and *A* is the surface area of the chamber (m^2^). If the *R* ^2^ value of the linear regression was <0.7, the GHG flux was removed from further statistical analysis. Modified from the upscaling method of Jeffrey et al.,^64^ we calculated the GHG flux per tree (*F*_t_; mg tree^–1^ h^–1^), stem surface area-weighted flux (*F*_ta_; mg m^–2^ stem h^–1^), and forest area-weighted stem flux (*F*_taw_; kg ha^–1^ yr^–1^) using eqs 2, 3, and 4, respectively:

$$F_{t}=\sum_{x=1}^{n}F_{x}\times P_{x}\times H_{x}$$ 2

$$F_{\mathrm{ta}}=\frac{F_{t}}{A_{t}}$$ 3

$$F_{\mathrm{taw}}=\sum_{s=1}^{n}F_{t,s}\times d\times c$$ 4

where *P* is the perimeter of the stem at the chamber position (m), *H* is the height of the interval (m), *A*_t_ is the total stem area of the tree up to the highest sampling interval, *F*_t,s_ is the GHG flux per tree for each season *s*, *d* is the tree density at each site (tree ha^–1^), and *c* is the conversion factor from mg to kg and from per hour to per year. Similarly, the forest area-weighted soil flux (*F*_saw_; kg ha^–1^ year^–1^) was calculated by scaling up the average soil GHG flux for each season from each site. The total ecosystem flux was subsequently calculated by summing *F*_taw_ and *F*_saw_. The total stem CO_2_e (CO_2_-equivalent) flux was calculated based on the GWP over a 100-year time scale (GWP_100_) using eq 5:

$$\mathrm{total}\;\mathrm{stem}\;{\mathrm{CO}}_{2}\;e\;\mathrm{fluxes}=\left(\left(F_{\mathrm{taw}\cdot {\mathrm{CH}}_{4}}\times {\mathrm{GWP}}_{{\mathrm{CH}}_{4}}\times c\right)+\left(F_{\mathrm{taw}\cdot N_{2}O}\times {\mathrm{GWP}}_{N_{2}O}\times c\right)\right)\times A_{f}$$ 5

where *F*_taw_ represents the forest area-weighted stem CH_4_ or N_2_O flux calculated using eq 4, GWP is GWP_100_ from the IPCC AR6 report (CH_4_: 27; N_2_O: 273), *c* is the conversion factor from kg to tonnes (0.001), and *A*_f_ is the area of the mangroves at each site (ha).

### Environmental Parameters

In the field, we measured the soil oxidation–reduction potential (ORP) and temperature within the soil chamber at the soil surface and at depths of 5 and 10 cm below the surface using a redox potential meter (ORP30, CLEAN L’eau, Taoyuan, Taiwan) on 10 cm soil cores extracted with a stainless-steel corer (7 cm in diameter). We then homogenized and stored the soil cores. Syringes (2.9 cm in diameter) were used to collect the top 5 cm of the soil for two subsamples within the soil chamber. These samples were stored in a portable cooler and transported to the laboratory for further analysis. The 10 cm soil core was used to determine the pH, salinity, and sulfate concentration. Specifically, we measured the pH and salinity using a pH meter (WD-35634-40; Oakton, Illinois, USA) and a salinity meter (Cond 3310; WTW, Germany) after the soil samples were diluted with double-distilled water at a 1:5 ratio. The two 5 cm soil samples were used to determine (i) soil mass wetness, organic matter content, and dry bulk density and (ii) medium grain size, silt–clay content, and sorting coefficient.^76–79^ Wood-specific density and moisture content were determined at the end of the study using tree cores extracted from each sampling tree at K-WZW, K-XF, A-FY, and A-BM.^80,81^ The cores were taken at intervals of 0–85 and 85–200 cm.

### Modeling Approach

Partial least-squares structural equation modeling (PLS-SEM) was selected for this study to predict and explain key constructs while testing a theoretical framework. PLS-SEM is effective for small sample sizes and large data sets and does not require a normal distribution, making it suitable for non-normally distributed data.^82^ Its causal–predictive nature balances predictive machine learning with confirmatory methods, making it valuable for research that seeks actionable recommendations.^83^ However, PLS-SEM has been underutilized in ecological studies, with few studies meeting the established standards.^84^

In the PLS-SEM, all of the constructs were measured reflectively. Specifically, the exogenous construct PHYSIC was measured reflectively using the following indicator variables: soil mass wetness, soil dry bulk density, median grain size, sorting coefficient, and silt and clay content. The exogenous construct TEMP and the endogenous construct REDOX were measured reflectively using soil temperature and ORP, respectively, at the soil surface and at depths of 5 and 10 cm below the surface. The endogenous construct TREE was measured reflectively using the following indicator variables: lenticel density, stem perimeter, and pneumatophore density (only in *A. marina*). The soil CH_4_ flux, soil N_2_O flux, stem CO_2_ flux, stem CH_4_ flux, and stem N_2_O flux served as indicators for the SCH4, SN2O, TCO2, TCH4, and TN2O constructs, respectively. The indicator variables were further filtered based on the assessment of the measurement model.^82,85^ First, the degree of variance explained by each indicator’s construct was indicated by its reliability, which is the square of the indicator loading. Therefore, indicators with loadings of less than 0.400 were eliminated from the measurement model. Indicators with loadings between 0.400 and 0.708 were considered for removal, while those with loadings greater than 0.708 were retained in the model. The internal consistency reliability, which measures the extent to which indicators of the same construct are associated with each other, was evaluated using the reliability coefficient (rho_A_), composite reliability (rho_C_), and Cronbach’s alpha. Constructs with internal consistency reliability values of less than 0.60 were eliminated from the model. Convergent validity was further evaluated using the average variance extracted (AVE); each construct should have an AVE ≥0.50. Finally, discriminant validity was assessed through the heterotrait–monotrait ratio (HTMT) criterion by ensuring that the upper boundaries of the 90% confidence intervals were smaller than 0.90. We ensured this by using bootstrap confidence intervals, employing 10,000 bootstrap subsamples, and setting the significance level at 0.10.^82,86^

For the structural model, the variance inflation factor (VIF) values were calculated to examine potential collinearity issues between the constructs, with values above 5 possibly indicating collinearity problems. The root-mean-square error (RMSE) values from the model were compared with those from the linear regression model (LM) for TCO2, TCH4, and TN2O to assess the model’s predictive power. Finally, structural models were compared across different configurations, and the model that minimized the Bayesian information criterion (BIC) was chosen for this study. The model was further computed by bootstrapping 10,000 subsamples and setting the significance level at 0.05, which produced the values and significance of the path coefficients as well as the *R* ^2^ of each construct.

### Statistical Analysis

All the statistical analyses were performed using R statistical software (v4.2.2).^87^ The Kruskal–Wallis test on ranks was used to evaluate the differences in GHG fluxes among species and the differences in environmental parameters among sites using the stats R package (v4.2.2). Dunn’s multiple comparison test was applied as a post hoc analysis using the dunn.test R package (v1.3.5) when the differences were significant. The differences between the GHG fluxes from the stem and soil surface were analyzed using the Wilcoxon rank sum test with the stats R package (v4.2.2). The results were considered to be statistically significant when the *p* value was less than 0.05. Plots were created mainly with the ggplot2 R package (v3.5.1),^88^ and the color palette was derived from the see R package (v0.11.0).^89^ The PLS-SEM was constructed using the seminr R package (v2.3.4).^82^ Data are presented primarily as the mean ± the standard deviation (SD).

## Results

### Environmental Parameters

Soil ORP generally decreases with depth, with L-AGH being an exception as it maintains relatively constant ORP values from the soil surface (0 cm) to a depth of 10 cm (Table 2). A-FY and A-BM exhibited negative ORP values, indicating a favorable environment for reducing reactions, whereas the other sites showed more oxidizing conditions. The average soil temperature ranged from 24.77 to 28.28 °C at 0 cm, from 24.29 to 28.17 °C at 5 cm, and from 24.08 to 28.02 °C at 10 cm, showing a slight decrease in temperature with increasing depth.

**Table 2:** Soil Environmental Parameters across All of the Studied Sites on the Western Coast of Taiwan^a^

	ORP (mV)			temperature (°C)											
site	0 cm	5 cm	10 cm	0 cm	5 cm	10 cm	mass wetness (%)	dry bulk density (g cm^–3^)	organic matter content (%)	pH^b^	salinity	sulfate concentration (g L^–1^)	median grain size (mm)	sorting coefficient	silt and clay content (%)
K-WZW	150.1 ± 72.75^b^	33.45 ± 74.94^d^	15.38 ± 76.08^d^	24.77 ± 4.79^c^	24.29 ± 4.56^c^	24.08 ± 4.40^c^	40.23 ± 8.43^b^	1.46 ± 0.16^cd^	5.22 ± 1.64^bf^	7.24 ± 0.36^ac^	1.13 ± 0.71^d^	0.76 ± 0.42^d^	0.22 ± 0.04^d^	1.34 ± 0.25^ac^	13.54 ± 3.74^b^
K-XF	182.35 ± 78.27^b^	129.92 ± 95.48^b^	136.65 ± 95.20^b^	25.75 ± 4.96^ac^	25.22 ± 4.74^ac^	24.83 ± 4.53^ac^	55.52 ± 9.71^c^	1.40 ± 0.12^d^	6.59 ± 1.78^cf^	6.37 ± 0.39^e^	1.59 ± 0.84^cd^	0.94 ± 0.92^d^	0.13 ± 0.03^b^	2.02 ± 0.18^d^	28.18 ± 5.13^c^
A-FY	0.68 ± 83.79^c^	–83.13 ± 74.74^c^	–96.23 ± 84.03^c^	25.96 ± 4.08^ac^	25.76 ± 4.00^ac^	25.63 ± 3.94^ac^	48.64 ± 10.53^bc^	1.58 ± 0.16^c^	3.54 ± 0.62^d^	7.90 ± 0.22^d^	3.71 ± 1.08^b^	1.93 ± 0.58^c^	0.04 ± 0.01^a^	1.08 ± 0.30^b^	82.81 ± 14.34^a^
A-BM	–14.71 ± 110.55^c^	–107.74 ± 130.83^c^	–126.61 ± 136.89^c^	26.63 ± 4.87^abc^	26.48 ± 4.83^ab^	26.37 ± 4.83^ab^	61.53 ± 15.81^c^	1.46 ± 0.21^cd^	3.90 ± 0.65^ad^	6.87 ± 0.53^b^	3.60 ± 1.04^b^	1.93 ± 0.62^c^	0.07 ± 0.03^c^	1.83 ± 0.20^d^	48.09 ± 13.64^c^
L-AGL	158.53 ± 86.04^b^	111.44 ± 88.18^b^	99.42 ± 72.09^b^	27.46 ± 4.25^abc^	28.17 ± 7.95^ab^	27.22 ± 4.17^ab^	43.27 ± 13.31^b^	0.66 ± 0.27^b^	16.25 ± 15.00^c^	6.89 ± 0.75^bc^	2.54 ± 1.86^a^	13.03 ± 5.58^b^	0.11 ± 0.03^b^	1.26 ± 0.43^bc^	18.65 ± 11.07^b^
L-AGH	238.49 ± 40.21^a^	236.73 ± 35.33^a^	236.73 ± 38.51^a^	27.81 ± 3.43^ab^	27.57 ± 3.27^ab^	27.33 ± 3.29^ab^	23.03 ± 5.88^a^	0.99 ± 0.25^a^	5.65 ± 2.98^ab^	7.45 ± 0.43^a^	2.33 ± 1.11^a^	6.41 ± 4.02^a^	0.03 ± 0.01^a^	1.49 ± 0.16^a^	71.49 ± 8.19^a^
R-HML	73.56 ± 89.37^d^	39.75 ± 91.85^d^	38.64 ± 87.84^d^	28.28 ± 5.57^b^	28.13 ± 5.46^b^	28.02 ± 5.32^b^	24.86 ± 5.94^a^	1.34 ± 0.2^3d^	2.26 ± 1.45^e^	6.27 ± 1.00^e^	2.01 ± 0.81^ac^	2.45 ± 2.67^c^	0.13 ± 0.04^b^	1.23 ± 0.41^c^	16.32 ± 8.17^b^

a Lowercase letters indicate significant differences among sites as determined by Dunn’s multiple comparison test.

b The pH and salinity were measured after diluting 5 g of the soil samples with 25 g of double-distilled water.

K-XF and A-BM presented the greatest soil mass wetness, averaging 55.52 and 61.53%, respectively, whereas L-AGH and R-HML presented the lowest mass wetness, averaging 23.03 and 24.86%, respectively (Table 2). Dry bulk density varied across sites, with values ranging from 0.66 g cm^–3^ at L-AGH to 1.58 g cm^–3^ at A-FY. The organic matter content ranged from 2.26% at R-HML to 16.25% at L-AGL. The pH values ranged from 6.27 at R-HML to 7.90 at A-FY, suggesting a generally neutral to slightly acidic soil environment. The salinity levels varied among the sites, with the highest recorded at K-XF (6.37 g L^–1^) and the lowest recorded at L-AGH (2.33 g L^–1^). Sulfate concentrations ranged from 0.76 g L^–1^ at K-XF to 3.71 g L^–1^ at A-FY.

The median soil grain size averaged 0.10 mm across all of the sites (Table 2). According to the USDA classification system, the soil texture at A-BM was very fine sand, whereas that at A-FY and L-AGH was silt. Fine sand was found at the remaining sampling sites. The sorting coefficient varied from 1.08 at A-FY to 2.02 at K-XF. Finally, the silt and clay content varied significantly, with A-FY showing the highest percentage (82.81%) and K-WZW showing the lowest percentage (13.54%).

### Soil and Stem GHG Flux

At the species level, the stems of all of the mangrove tree species acted as sources of CO_2_, CH_4_, and N_2_O, with significant variation among species (Figure 2). The mean stem CO_2_ fluxes of *A. marina*, *L. racemosa*, *R. stylosa*, and *K. obovata* were 234.71 ± 222.21, 51.66 ± 36.95, 34.57 ± 64.71, and 21.87 ± 26.36 mg m^–2^ h^–1^, respectively. *A. marina* exhibited significantly greater CH_4_ flux, averaging 61.56 ± 149.47 μg m^–2^ h^–1^, while there was no significant difference between *R. stylosa* (5.77 ± 26.48 μg m^–2^ h^–1^), *K. obovata* (0.93 ± 3.06 μg m^–2^ h^–1^), and *L. racemosa* (0.14 ± 2.14 μg m^–2^ h^–1^). The stem N_2_O flux followed a similar trend, with a significantly greater flux found in *A. marina* (521.04 ± 612.41 μg m^–2^ h^–1^), followed by *L. racemosa* (1.71 ± 3.46 μg m^–2^ h^–1^), *R. stylosa* (1.27 ± 3.02 μg m^–2^ h^–1^), and *K. obovata* (0.49 ± 0.96 μg m^–2^ h^–1^). However, the GHG flux from the stem surface was significantly lower than that from the soil, except for the N_2_O flux in *A. marina*. Specifically, the N_2_O emissions from the stem surface of *A. marina* were 67 times higher than those from the soil surface (7.84 ± 25.93 μg m^–2^ h^–1^). In contrast, the soil N_2_O fluxes were approximately 10 to 29 times greater than those from the stems of *L. racemosa*, *R. stylosa*, and *K. obovata*, averaging 49.08 ± 139.28, 12.76 ± 27.14, and 12.61 ± 35.84 μg m^–2^ h^–1^, respectively. The soil CH_4_ flux was significantly greater in *A. marina* (1850.50 ± 6745.64 μg m^–2^ h^–1^), followed by *K. obovata* (862.67 ± 3829.89 μg m^–2^ h^–1^), *L. racemosa* (340.38 ± 1304.45 μg m^–2^ h^–1^), and *R. stylosa* (72.40 ± 174.89 μg m^–2^ h^–1^), whose values were 30 to 2443 times greater than the average stem CH_4_ flux (Figure 2). The soil CO_2_ flux did not significantly differ among species, with the average flux ranging from 118.27 to 477.63 mg m^–2^ h^–1^.

**Figure 2:**
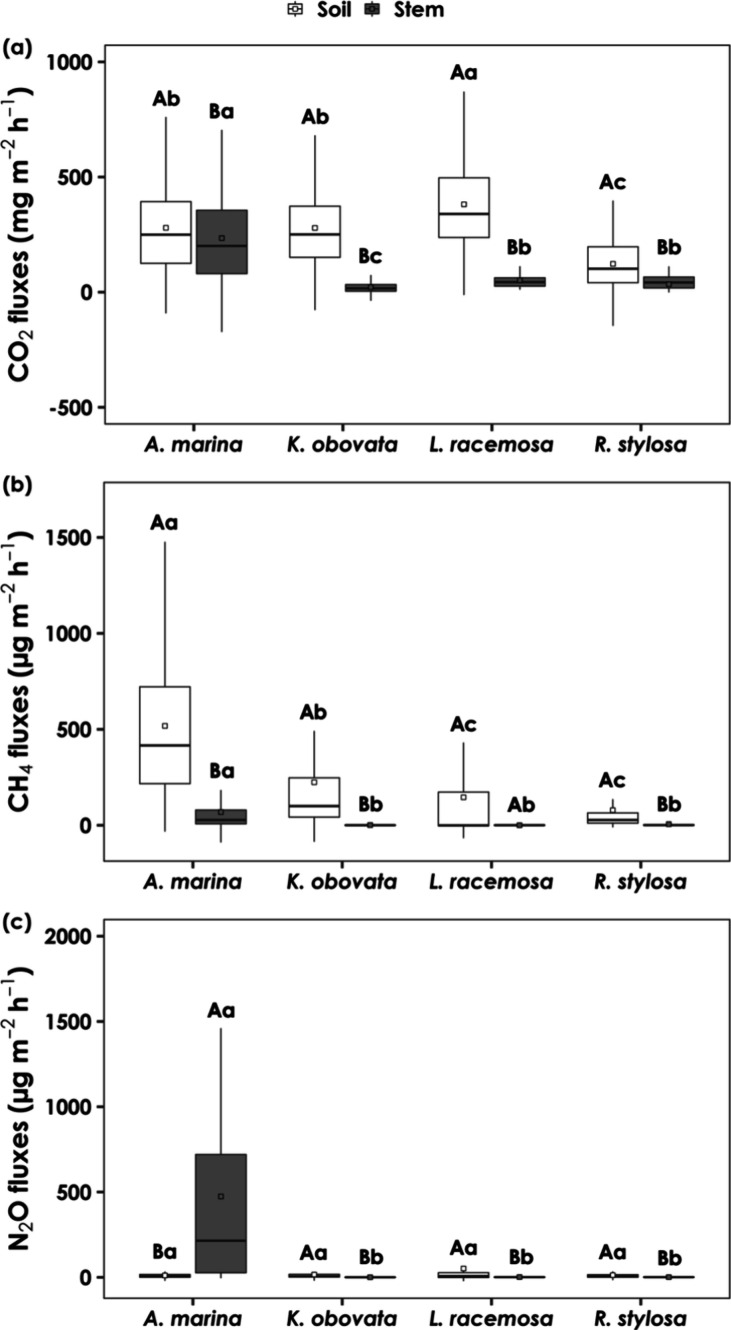
Soil and stem fluxes of (a) carbon dioxide (CO_2_), (b) methane (CH_4_), and (c) nitrous oxide (N_2_O) for each studied species. The stem fluxes presented were stem surface area-weighted fluxes (*F*_ta_). The square in the box plot represents the average value. Significant differences between stem and soil GHG fluxes are indicated by uppercase letters, while significant differences between species are indicated by lowercase letters. Outliers (defined as values falling below Q1–1.5 * IQR or above Q3 + 1.5 * IQR) were removed for easier interpretation.

At the site level, the forest area-weighted stem CH_4_ flux of *L. racemosa* at L-AGL averaged −0.03 kg ha^–1^ year^–1^, making it the only stem-mediated CH_4_ sink (Table 3). The average forest area-weighted stem CH_4_ flux at the remaining sites ranged from 0.01 to 4.19 kg ha^–1^ year^–1^. The contribution of stem CH_4_ flux to the total ecosystem CH_4_ flux was generally low, accounting for less than 5%. In contrast, the stem N_2_O flux of *A. marina* contributed approximately 100% to the total ecosystem N_2_O fluxes at A-FY and A-BM, completely offsetting the N_2_O sink capacity of the soil at A-BM. At L-AGH, the stem N_2_O flux contributed 52% because of low soil N_2_O emissions, whereas the contribution of the stem ranged from 1.19 to 3.85% at the remaining sites. In terms of the soil carbon burial rate, L-AGH presented the highest rate at 332.37 t CO_2_e year^–1^, followed by A-FY at 65.04 t CO_2_e year^–1^. The soil carbon burial rates at the remaining sites ranged from 0.26 t CO_2_e year^–1^ to 48.61 t CO_2_e year^–1^. However, the soil carbon burial was completely offset by the total stem CO_2_e flux in *A. marina*, with offsets of 889.28% at A-BM and 313.31% at A-FY. In contrast, the offset by total stem CO_2_e flux was less than 5% in *K. obovata*, *L. racemosa*, and *R. stylosa*.

**Table 3:** Forest Area-Weighted Stem and Soil CH_4_ and N_2_O Fluxes at the Studied Sites on the Western Coast of Taiwan

site	species	forest area-weighted stem CH_4_ flux (kg ha^–1^ year^–1^)	total ecosystem CH_4_ flux (kg ha^–1^ year^–1^)^a^	forest area-weighted stem N_2_O flux (kg ha^–1^ year^–1^)	total ecosystem N_2_O flux (kg ha^–1^ year^–1^)^a^	carbon burial rate (t CO_2_e year^–1^)^c^	total stem CO_2_e flux (t CO_2_e year^–1^)^b^	offset (%)^d^
K-WZW	*Kandelia obovota*	0.05	101.44	0.05	1.21	48.61	0.14	0.29
K-XF	*Kandelia obovota*	0.07	16.60	0.04	1.09	42.28	0.11	0.26
A-FY	*Avicennia marina*	2.45	140.29	24.15	25.45	65.04	64.52	889.28
A-BM	*Avicennia marina*	4.19	159.18	42.56	42.51	7.26	203.77	313.31
L-AGL	*Lumnitzera racemosa*	–0.03	73.42	0.11	6.81	33.52	0.55	1.63
L-AGH	*Lumnitzera racemosa*	0.01	–0.33	0.05	0.10	332.37	0.63	0.19
R-HML	*Rhizophora stylosa*	0.06	6.42	0.01	1.16	0.26	0.003	1.22

a The total ecosystem flux was calculated by summing *F*_taw_ and *F*_saw_, as shown in eq 4.

b The total stem CO_2_e flux was calculated by summing the converted CH_4_ and N_2_O fluxes using the global warming potential (GWP) over 100 years from the IPCC AR6 report.

c The carbon burial rate was sourced from Lin et al. and unpublished data.^23^ The carbon burial was converted from carbon to CO_2_e using the ratio of the molar mass of CO_2_ to that of carbon.

d The offset percentage was calculated by dividing the total stem CO_2_e flux by the carbon burial rate.

### Vertical Patterns

Although significant differences between height intervals were detected mainly in *A. marina* (Table S1), the stem CH_4_ flux tended to be higher at the lowest stem heights (Figure 3). Specifically, the stem CH_4_ flux was highest at 0–40 cm aboveground in *A. marina* and *K. obovata*, averaging 156.96 ± 451.22 and 4.30 ± 12.04 μg m^–2^ h^–1^, respectively (Figure 3). This accounted for more than 50% of the CH_4_ flux per tree (*F*_t_). In *K. obovata*, the stems at height intervals of 85–140 and 140–200 cm aboveground even served as net CH_4_ sinks, with fluxes averaging −1.50 ± 9.87 and −0.12 ± 7.57 μg m^–2^ h^–1^, respectively. The stem CH_4_ flux was also greater at 0–40 cm aboveground in *R. stylosa*, averaging 8.93 ± 48.65 μg m^–2^ h^–1^, compared with that at 40–80 cm aboveground (1.96 ± 3.55 μg m^–2^ h^–1^). Interestingly, the highest CH_4_ flux in *L. racemosa* occurred at the highest stem height, at 90–120 cm aboveground, with an average value of 0.7 ± 1.44 μg m^–2^ h^–1^. In contrast, the stem surfaces at 30–60 and 60–90 cm aboveground served as net CH_4_ sinks, with average values of −0.08 ± 5.47 and −0.05 ± 3.86 μg m^–2^ h^–1^, respectively.

**Figure 3:**
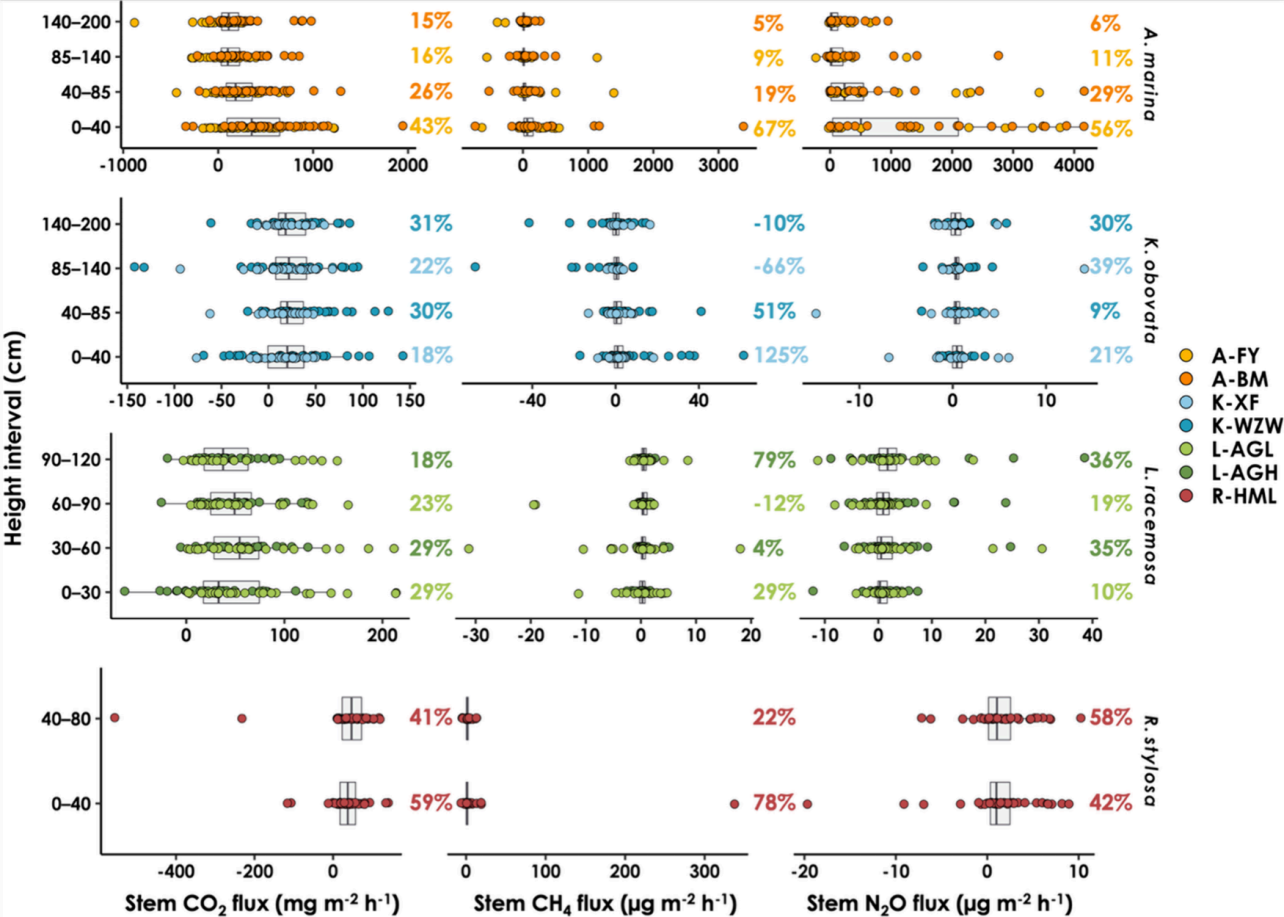
Vertical pattern of stem carbon dioxide (CO_2_), methane (CH_4_), and nitrous oxide (N_2_O) fluxes. The percentage value on the right side of each subplot represents the contribution of the stem GHG flux at each height interval to the GHG flux per tree (*F*_t_; mg tree^–1^ h^–1^). A negative contribution indicates that the direction of the flux at that specific height interval differs from that of *F*_t_. Note that the scale of the *x*-axis is different for each subplot.

The stem N_2_O flux remained positive across the different stem heights among all the studied species, showing no consistent pattern, except for *A. marina*, where the stem N_2_O flux was highest at 0–40 cm above ground (1181.66 ± 1349.94 μg m^–2^ h^–1^), followed by 40–85 cm (616.77 ± 980.48 μg m^–2^ h^–1^), 85–140 cm (220.48 ± 520.86 μg m^–2^ h^–1^), and 140–200 cm aboveground (133.10 ± 225.04 μg m^–2^ h^–1^). The contribution of stem N_2_O at the highest height interval was only 6% of the N_2_O flux per tree in *A. marina*. In contrast, the contribution at the highest height interval was at least 30% in *K. obovata*, *L. racemosa*, and *R. stylosa* (Figure 3).

### Seasonal Variations

Stem CH_4_ flux exhibited significant seasonal variations (Figure 4). In *A. marina*, the stem CH_4_ flux was highest in the summer at A-FY (114.09 ± 146.72 μg m^–2^ h^–1^) and in the autumn at A-BM (127.11 ± 351.99 μg m^–2^ h^–1^). The lowest flux occurred in the winter at both sites (A-FY: 4.56 ± 9.38 μg m^–2^ h^–1^; A-BM: 8.04 ± 16.46 μg m^–2^ h^–1^). The stem CH_4_ fluxes in *K. obovata* showed a similar trend, peaking in the autumn at K-XF (2.08 ± 2.64 μg m^–2^ h^–1^) and in the summer at K-WZW (3.00 ± 5.73 μg m^–2^ h^–1^). However, the lowest CH_4_ flux in K-WZW occurred during spring, with an average value of −0.66 ± 2.12 μg m^–2^ h^–1^. At K-XF, the lowest flux was measured in the winter, emitting negligible amounts of CH_4_ (0.05 ± 0.24 μg m^–2^ h^–1^). Interestingly, the stem CH_4_ fluxes of *L. racemosa* displayed different seasonal trends. The highest CH_4_ flux at L-AGL occurred in the spring (0.87 ± 2.19 μg m^–2^ h^–1^). Additionally, it acted as a CH_4_ sink during the autumn, despite considerable variations, with an average of −3.24 ± 5.45 μg m^–2^ h^–1^. This also caused the tree stem of *L. racemosa* at L-AGL to become a net CH_4_ sink annually (Table 3). At L-AGH, the highest and lowest stem CH_4_ fluxes were observed in the summer (0.73 ± 1.02 μg m^–2^ h^–1^) and autumn (0.14 ± 0.09 μg m^–2^ h^–1^), respectively. *R. stylosa* exhibited peak CH_4_ fluxes in the summer (16.37 ± 52.75 μg m^–2^ h^–1^) and the lowest in the spring (0.40 ± 1.32 μg m^–2^ h^–1^). Overall, the stem CH_4_ fluxes during summer contributed the most to the forest area-weighted stem CH_4_ flux (*F*_taw_), averaging 50.94 ± 46.23% across all of the sites. This was followed by autumn at 49.86 ± 72.71%, winter at 5.55 ± 15.88%, and spring at −6.35 ± 32.27%. The soil CH_4_ flux showed a similar pattern, peaking in summer and reaching its lowest point in winter (Figure S2).

**Figure 4:**
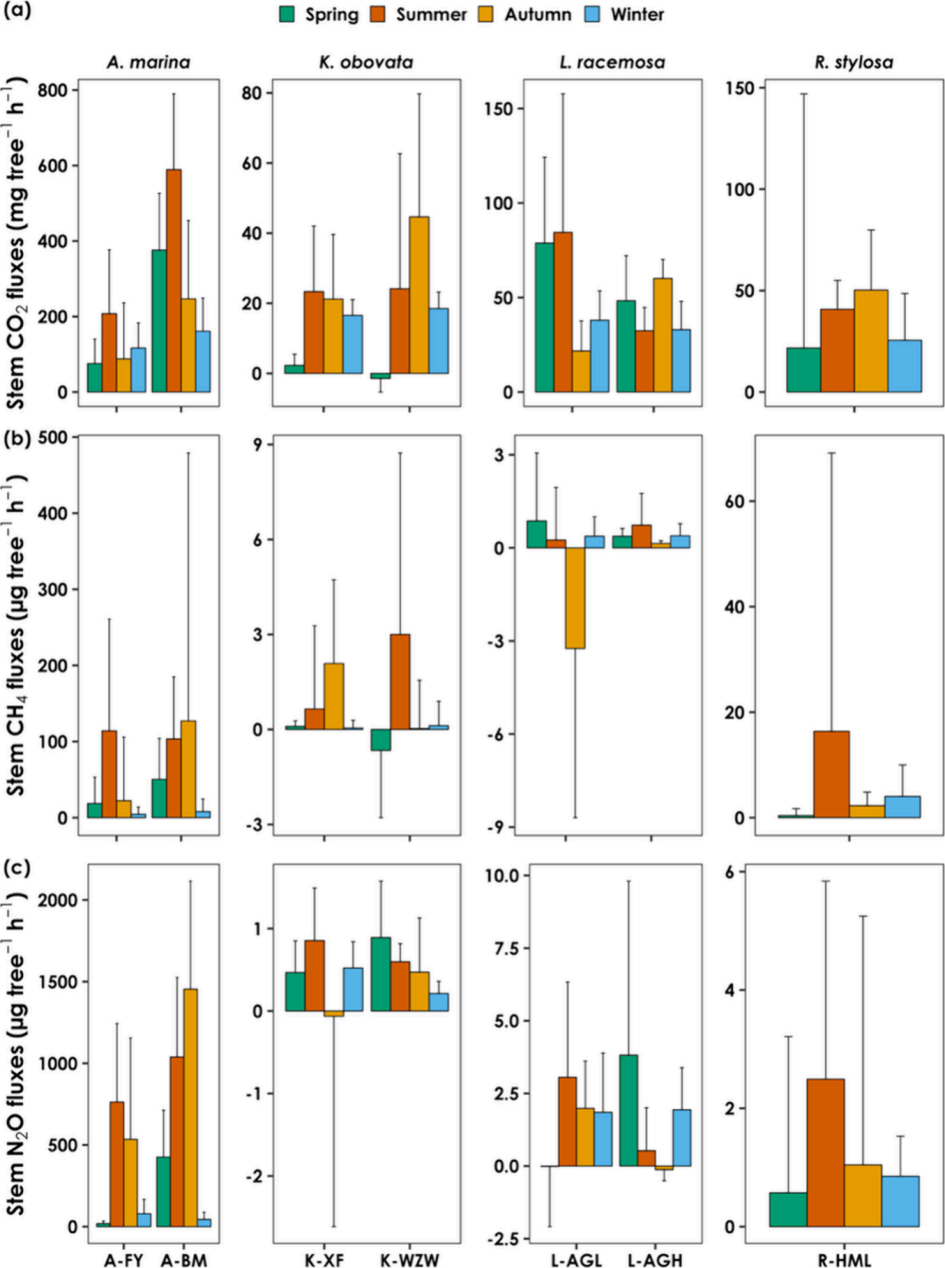
Seasonal variations in stem-mediated (a) carbon dioxide (CO_2_), (b) methane (CH_4_), and (c) nitrous oxide (N_2_O) fluxes for each studied species. Spring, summer, autumn, and winter are defined as March to May, June to August, September to November, and December to February, respectively. Note that the scale of the *x*-axis is different in each subplot.

In contrast, the stem N_2_O fluxes did not exhibit a consistent seasonal pattern (Figure 4). Overall, the contributions were highest in summer (39.21 ± 16.51%), followed by spring (22.16 ± 23.15%) and autumn (21.51 ± 20.18%), and lowest in winter (17.11 ± 11.72%) across all of the sites. Among the sites, the tree stems of *A. marina* exhibited higher N_2_O fluxes in autumn and summer. At A-FY, the average stem N_2_O flux was 762.83 ± 480.22 μg m^–2^ h^–1^ in the summer and 534.81 ± 620.34 μg m^–2^ h^–1^ in autumn. At A-BM, the average stem N_2_O flux was 1038.59 ± 487.10 μg m^–2^ h^–1^ in the summer and 1454.38 ± 661.24 μg m^–2^ h^–1^ in autumn. Conversely, the stem N_2_O fluxes of *L. racemosa* were greater in the spring and winter at L-AGH, averaging 3.82 ± 5.99 and 1.94 ± 1.44 μg m^–2^ h^–1^, respectively. However, the stem N_2_O flux did not significantly differ across seasons in K-WZW, K-XF, L-AGL, and R-HML (*p* > 0.05). Moreover, the stem acted as a weak N_2_O sink during the autumn in K-XF (−0.06 ± 2.55 μg m^–2^ h^–1^) and L-AGH (−0.13 ± 0.38 μg m^–2^ h^–1^), as well as during the spring in L-AGL (−0.02 ± 2.07 μg m^–2^ h^–1^). Similar to stem N_2_O fluxes, soil N_2_O fluxes did not show a consistent seasonal pattern (Figure S2).

### Structural Equation Model

The path coefficients (β), derived from bootstrapping with 10,000 subsamples, revealed significant relationships among the latent constructs. Among the exogenous constructs, TEMP plays the most prominent role in the model (Figure 5). In all the studied species except *R. stylosa*, TEMP had a direct positive effect on TCO2, with the path coefficient ranging from 0.22 to 0.44 (*p* < 0.05). TEMP also positively affected TCH4 in *A. marina* (β = 0.21, *p* < 0.001) and *R. stylosa* (β = 0.44, *p* < 0.001). In *A. marina*, TEMP had a positive effect on SN2O (β = 0.32, *p* < 0.001) and TN2O (β = 0.53, *p* < 0.001). Interestingly, TEMP positively influenced TCH4 (β = 0.19, *p* < 0.05) in *K. obovata* but negatively affected TCH4 in *L. racemosa* (β = −0.18, *p* < 0.05) and *R. stylosa* (β = −0.19, *p* < 0.05). Indirect effects were also observed between TEMP and TCH4. In *L. racemosa*, TEMP indirectly affected TCH4 through the direct effect of TCO2 on TCH4 (β = 0.34, *p* < 0.001). Similarly, in *R. stylosa*, TEMP also indirectly affected TCH4 through the direct effect of TREE on TCH4 (β = −0.20, *p* < 0.51) and through the direct effect of SCH4 on TCH4 (β = 0.75, *p* < 0.01).

**Figure 5:**
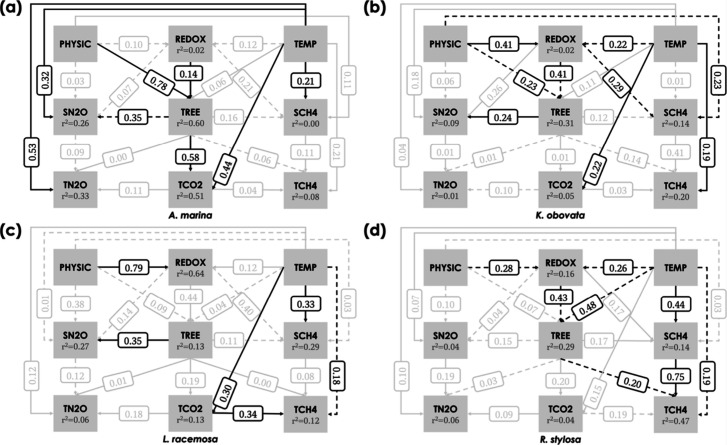
Partial least-squares structural equation model (PLS-SEM) of (a) *Avicennia marina*, (b) *Kandelia obovata*, (c) *Lumnitzera racemosa*, and (d) *Rhizophora stylosa*. Only the inner models are shown. The values shown by the arrows connecting latent variables represent path coefficients. A black arrow indicates a significant direct effect, while a gray arrow indicates a nonsignificant effect. The solid lines and dashed lines represent positive and negative relationships, respectively.

In addition to TEMP, the exogenous construct PHYSIC had direct positive effects on TREE and indirect positive effects on TCO2 through TREE in *A. marina*. PHYSIC also significantly affected REDOX in all the studied species except *A. marina*, with path coefficients ranging from −0.28 to 0.79 (*p* < 0.01). In *K. obovata*, PHYSIC had a negative effect on TREE (β = −0.23, *p* < 0.05) and SCH4 (β = −0.23, *p* < 0.05). Among the endogenous constructs, REDOX positively affected TREE in *A. marina* (β = 0.14, *p* < 0.05) and negatively affected SN2O in both *R. stylosa* (β = −0.43, *p* < 0.001) and *K. obovata* (β = −0.41, *p* < 0.001). REDOX also had a negative effect on SCH4 in *K. obovata* (β = −0.29, *p* < 0.01). Subsequently, TREE negatively affected SN2O in *A. marina* (β = −0.35, *p* < 0.001) and positively affected SN2O in both *L. racemosa* (β = 0.24, *p* < 0.05) and *K. obovata* (β = −0.35, *p* < 0.05).

The key target constructs in this study are TCO2, TCH4, and TN2O. The model explained 51% of the variance in TCO2 for *A. marina*, as indicated by the *R* ^2^ value. In contrast, the models for the other studied species demonstrated weaker explanatory power for TCO2, with *R* ^2^ values ranging from 0.04 to 0.13. Similarly, the *R* ^2^ value for TN2O was highest in the model for *A. marina* at 0.33, while the other models explained less than 10% of the variance in TN2O. For TCH4, the highest *R* ^2^ value was observed for *R. stylosa* (0.47), followed by *K. obovata* (0.20), *L. racemosa* (0.12), and *A. marina* (0.08). Despite the low *R* ^2^ value, the model demonstrated high predictive power, as evidenced by a lower RMSE compared to the LM benchmark (Table S2). Specifically, the indicators for TCO2, TCH4, and TN2O of *A. marina* had RMSE values of 162.93, 0.161, and 0.387, respectively, in the PLS-SEM. In contrast, the corresponding values in the LM were 209.109, 0.171, and 0.426, respectively. The trend was also observed in other studied species.

## Discussion

### Spatial and Species Variations in GHG Fluxes

This study revealed significant variations in both stem and soil CH_4_ fluxes among mangrove species. Specifically, compared with the other studied species, *A. marina* presented significantly greater stem and soil CH_4_ fluxes (Figure 2). The pneumatophores of *A. marina* were not intentionally excluded from the chamber measurements in this study. Although pneumatophores do not always promote CH_4_ production,^63^ their presence likely acted as an additional conduit for CH_4_ emissions, resulting in higher soil CH_4_ fluxes in *A. marina*.^29–31^ Similarly, Zhang et al. reported that the pneumatophores of *A. marina* exhibited significantly higher CH_4_ fluxes (512.61 ± 426.40 μg m^–2^ h^–1^) compared to the soil (28.74 ± 15.37 μg m^–2^ h^–1^) and tree stem (44.78 ± 34.08 μg m^–2^ h^–1^).^67^ This explains the significantly higher soil CH_4_ fluxes compared to stem CH_4_ fluxes in *A. marina* in this study (Figure 2). However, previous studies have demonstrated that the stem CH_4_ flux from *K. obovata* and *Aegiceras corniculatum*, which lack pneumatophores, is greater than that from *A. marina*.^62,67^ This contradicts the results of this study, which revealed the highest stem CH_4_ fluxes in *A. marina* (Figure 2). Furthermore, He et al. reported that the stem of *Kandelia candel* consumed CH_4_ at a rate of −29.00 ± 16.00 μg m^–2^ h^–1^, whereas the average stem CH_4_ fluxes ranged from −7.90 to 42.00 μg m^–2^ h^–1^ in three other mangrove species with pneumatophores.^63^ This finding suggests that the presence of pneumatophores may not play an important role in affecting species variations in the stem CH_4_ flux.

As mangroves often develop aerenchyma tissue to facilitate gas exchange,^44,90^ the tree stems of mangroves are also suggested to act as conduits for CH_4_ produced in the sediment.^64–67^ In this context, it is noteworthy that stem-mediated gas flux generally decreases with increasing stem height,^53,60^ a trend observed in CH_4_ fluxes in *A. marina* (Figure 3). This suggests a belowground origin for CH_4_ emissions. However, CH_4_ consumption by mangrove stems has also been demonstrated.^61–63,66^ The ability of tree stems to oxidize CH_4_ is attributed to the presence of methanotrophs, which can coexist with methane-producing bacteria.^55,91,92^ In this study, *L. racemosa* acted as a net CH_4_ sink at L-AGL but served as a net CH_4_ source at L-AGH (Table 3). Moreover, CH_4_ absorption was also observed at greater stem heights in *A. marina* and *K. obovata* (Table S1). Gao et al. demonstrated that the stem of *K. obovata* functioned as both a CH_4_ sink and a source at different sites, which depended on CH_4_ production in the soil.^62^ Interestingly, this was not the case in our study. The soil at L-AGH presented higher CH_4_ emissions, while L-LGH acted as a weak CH_4_ sink (Table 3). Therefore, we speculated that the stem of *L. racemosa* at L-AGH may serve as a conduit for CH_4_ produced in deeper soil, bypassing the favorable environment for CH_4_ oxidation near the soil surface, as indicated by the positive soil ORP (Table 2). However, we did not find a significant relationship between soil and stem CH_4_ fluxes (Figure 5). This may be due to the temporal and spatial mismatch between CH_4_ production and emissions through the soil–tree–atmosphere continuum.^53,93,94^

Most research on stem-mediated N_2_O flux has focused on upland forests or other wetland ecosystems (Figure 6). To our knowledge, Liao et al. is the only study that reported the N_2_O flux from mangrove tree stems, which averaged 2.09 ± 0.21 and 2.05 ± 0.19 μg m^–2^ h^–1^ for *K. obovata* and *Sonneratia apetala*, respectively, falling within the range observed in this study.^65^ However, the stem N_2_O flux from *A. marina* in this study was among the highest reported in the literature (Figure 6). Specifically, only the stem N_2_O flux from *Simarouba amara* in tropical upland forests was comparable to our findings, averaging 1193 ± 361 μg m^–2^ h^–1^ at 0.3 m aboveground.^95^ Although the average flux was not reported by Machacova et al., mesocosm experiments revealed that *Alnus glutinosa* and *Fagus sylvatica*, when subjected to flooding, can exhibit peak fluxes (5846 μg m^–2^ h^–1^) comparable to the maximum stem N_2_O flux detected in *A. marina* in this study (4162.28 μg m^–2^ h^–1^).^96^ Despite the high stem N_2_O flux, the soil served as a weaker N_2_O source in *A. marina* (Figure 2 and Table 3). This finding aligns with that of Welch et al., who suggested that the N_2_O emitted from the stem originated from deeper soil layers.^95^ In *K. obovata* and *L. racemosa*, N_2_O consumption was observed during specific seasons (Figure 4). Cryptogamic cover on tree stems can contribute to N_2_O consumption.^97–99^ However, this pathway was not included in this study.

**Figure 6:**
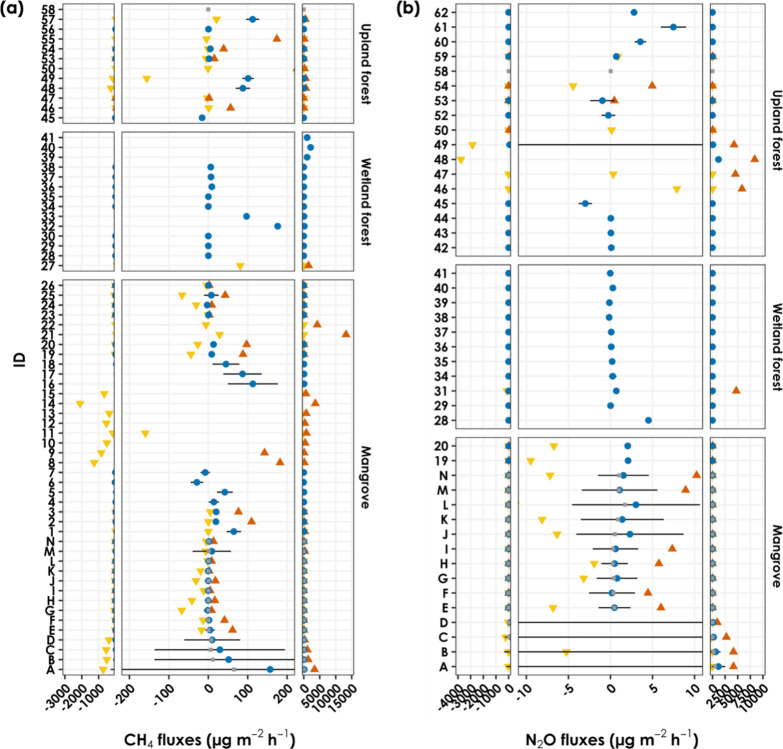
Comparison of stem CH_4_ and N_2_O fluxes among studies of mangroves, other wetland forests, and upland forests. The references corresponding to the IDs can be found in Table S3. Overall, IDs from A to N represent data from this study, while IDs from 1 to 62 present data from other related studies. A blue circle denotes the average value, a red square indicates the median, an error bar represents the standard deviation, a triangle marks the maximum value, and an inverted triangle signifies the minimum value. Please note that the study of Pangala et al.^50^ was excluded from this figure because of its extremely high value, which would affect readability.

### Temporal Variations Explained by Temperature Changes

To our knowledge, He et al. is the only study on mangroves that measured stem CH_4_ fluxes across all seasons, although no significant seasonal variation was observed.^63^ In contrast, this study revealed that stem CH_4_ fluxes were greater in summer or autumn, while winter fluxes had a minor effect on the upscaled flux (Figure 4 and Table 3). Jeffrey et al. reported that the stem CH_4_ flux of *A. marina* averaged 25.06 ± 3.88 μg m^–2^ h^–1^ during winter, which was approximately four times greater than the winter stem CH_4_ flux of *A. marina* in this study (6.14 ± 12.48 μg m^–2^ h^–1^).^64^ Additionally, this study is the first to capture the seasonal variations in stem N_2_O flux from mangrove tree stems (Figure 4). Similar seasonal trends were observed for both stem CH_4_ and N_2_O in wetlands and upland forests.^58,59,91,93,100,101^ Although fluctuations in temperature may significantly influence the seasonal variation in stem GHG flux,^43^ complex interactions among various environmental factors are likely more prominent.^100^ In this study, we observed both direct and indirect effects of the temperature on stem GHG flux across different mangrove species using PLS-SEM (Figure 5). Temperature positively affects stem CH_4_ and N_2_O in *K. obovata* and *A. marina*, respectively. This effect can be attributed to the increased activity of CH_4_- and N_2_O-producing bacteria, along with enhanced concentration-driven molecular diffusion and sap flow within the stem, depending on the sources of GHG.^43,100^ Interestingly, the indirect effect of the temperature on stem CH_4_ flux was mediated by stem CO_2_ flux in *L. racemosa* and by tree physiological traits in *R. stylosa* (Figure 5). Since stem CO_2_ flux is considered a reliable proxy for tree physiological activity, positive correlations were generally observed between stem CO_2_ flux and both stem CH_4_ and N_2_O flux.^58,93,102,103^ Therefore, these findings highlight the intricate interplay between environmental factors and tree physiological processes that drive the seasonal variations in the GHG flux from mangrove tree stems.

Stem diameter can adversely affect stem GHG flux because a larger diameter creates a greater barrier for the gases produced in the stem to reach the atmosphere.^59,104^ However, it can also be positively correlated because of the presence of aerenchyma tissue or differences in root morphology and biomass.^53,105–107^ On the other hand, stem lenticel density has a positive effect on stem GHG emissions, acting as a conduit to facilitate gas exchange between the stem and the atmosphere interface.^44,67,104,107–109^ Although not included in the PLS-SEM, the relationships between stem GHG flux and both wood-specific density and moisture content were also analyzed in *A. marina* and *K. obovata* (Figure S5). Previous studies have demonstrated that high wood-specific density may inhibit gas diffusion, while high wood moisture content may promote CH_4_ production by creating an anaerobic environment.^44,67,95,104,109^ However, in this study, no consistent pattern or significant relationship was observed (Figure S5), underscoring the need for further investigation into the relationship between wood properties and stem GHG flux.

In this study, GHG flux measurements were conducted during the daytime hours at low tide. However, based on research conducted at the same sites studied here containing *A. marina* and *K. obovata*, tidal influence on stem GHG flux in mangroves should also be considered.^66^ Specifically, the stem CH_4_ flux was 3.68% smaller at K-WZW when considering tidal influence, while it was 6.21 to 1200.25% larger at K-XF, A-FY, and A-BM. This variation may lead to an underestimation of the stem CH_4_ flux. Previous studies have also demonstrated a significant effect of water level on stem GHG flux.^110–113^ Although this study did not assess the impact of tidal influence, we compared *A. marina* and *R. stylosa* growing at sites with different inundation durations. Sites with longer inundation durations exhibited higher soil mass wetness and lower ORP (Figure S3), primarily because of the slower diffusion rate of oxygen in saturated sediment, which creates an anoxic environment.^44^ Additionally, these sites presented significantly higher soil CH_4_ and N_2_O fluxes (Figure S3). In contrast, stem GHG fluxes did not significantly differ between sites with different inundation durations (Figure S3). An anoxic environment caused by longer inundation favors CH_4_ production,^32,114^ thereby inducing higher CH_4_ emissions across the soil–atmosphere interface.^95,115,116^ The same is true for N_2_O production,^117–119^ suggesting that soil conditions under different inundation durations can significantly influence soil CH_4_ and N_2_O emissions without corresponding differences in stem GHG fluxes.

During the daytime, the transport mechanism of gases may shift from a slower rate of diffusion to a faster rate of convection, increasing the GHG emissions mediated by the stem.^120^ However, even if a diurnal pattern exists, radial transport is considered to have a more prominent effect.^53^ Therefore, stem CH_4_ and N_2_O fluxes mostly show either no significant difference or a consistent pattern in terms of diurnal variations.^100,102,121,122^ Although this study did not conduct nighttime measurements, we covered the chamber with opaque cloth to mimic a dark environment in *R. stylosa* and *L. racemosa* (Figure S4). In *R. stylosa*, stem CO_2_ and N_2_O fluxes were significantly greater under light conditions, while stem CH_4_ flux was significantly greater under dark conditions in *L. racemosa* (Figure S4). We speculate that this may be due to the effect of sunlight on the temperature inside the chamber or the cryptogamic organisms growing on the tree stem,^62,98,102,123^ which should be considered in future studies.

### Significance of Stem-Mediated Fluxes in Mangroves

When the GHG flux across entire ecosystems was compared, the contribution of tree stems varied significantly among the studies. Although soil typically acts as a CH_4_ sink in upland forests, emissions from stems can offset this capacity by up to 90%.^93,122^ In some cases, this can even shift ecosystems from being net sinks to sources of GHG.^124^ In wetlands, where soil CH_4_ fluxes are generally high,^30,75^ stem emissions can contribute as much to the total ecosystem CH_4_ flux as soil emissions.^67^ However, the contribution of stem CH_4_ flux was low compared with that of the soil in this study (Figure 1 and Table 3), which is consistent with the findings of other related studies.^59,63,102,116,125^ Previous studies often detected lower N_2_O emissions from stems than from soil.^96,126^ Even when both served as N_2_O sinks, the magnitude was shown to be lower for stems.^100^ In contrast, we found that N_2_O played an important role in the total ecosystem N_2_O flux, especially in *A. marina* and *L. racemosa* (Figure 1 and Table 3). Moreover, we measured stem GHG flux only up to 2 m aboveground, while tree heights can reach even greater levels (Table 1). Although stem GHG flux at greater heights could result in negligible emissions, the observed vertical pattern was not consistent across all species (Figure 3 and Table S1). This suggests that the upscaled stem flux in this study may have been underestimated. Furthermore, the canopy can contribute significantly to the ecosystem flux,^60,127^ which was also neglected in this study.

The unique environment of mangrove soils inhibits the decomposition of organic matter while promoting the storage of organic carbon.^19,128^ However, CH_4_ and N_2_O emissions from the soil may significantly offset the rate of soil carbon burial or have a minor effect.^34–36,129,130^ Previous studies conducted at the same sampling sites have determined the soil carbon burial rate in mangroves by subtracting detritus export and decomposition from litterfall production.^22,23^ Therefore, we converted the site-specific carbon burial rate to CO_2_e for each sampling site and assessed the CO_2_e flux of stem CH_4_ and N_2_O using GWP_100_ from the IPCC AR6 report. This allowed us to evaluate the effect of stem GHG flux on the carbon sequestration capacity of mangroves (Table 3). The soil carbon burial rate used in this study, which ranged from 0.109 to 203.77 t CO_2_e year^–1^, was lower than the global average.^20,131^ However, it accounts for only autochthonous sources of carbon and not allochthonous sources, which could lead to underestimation.^22,23^ Consequently, the results indicated that GHG emissions from mangrove tree stems can offset more than 100% of the carbon burial rate in *A. marina*, while the offset ranged from 0.19 to 1.63% in *K. obovata*, *L. racemosa*, and *R. stylosa* (Table 3). This emphasizes the need to include stem-mediated GHG emissions when assessing the overall carbon dynamics in mangrove ecosystems, particularly *A. marina*.

At the global scale, the CH_4_ and N_2_O budgets provide a comprehensive overview of the sources, both natural and anthropogenic, and sinks of GHGs in the atmosphere.^5,6^ This information allows policymakers to target specific mitigation efforts and supports the evaluation of national and global policies. However, significant discrepancies remain in the modeling approaches for global CH_4_ and N_2_O emissions.^47,48^ Moreover, data on GHG emissions from the VCEs across the soil–plant–atmosphere continuum are limited, leading to uncertainties in the budget.^5,6^ To address this, we estimated mangrove stem-mediated emissions on a global scale. We first gathered the most recent data on tree density and the area of global mangroves.^132,133^ We used the GHG flux per tree (*F*_t_) across all mangrove species in this study to calculate the median [Q1–Q3] fluxes, which we then multiplied by the tree density and area of global mangroves (Table 4). This resulted in global mangrove stem-mediated emissions of 0.006 [0.001–0.169] Gg CH_4_ year^–1^ and 0.009 [0.002–0.036] Gg N_2_O year^–1^ (Table 4). The uncertainty associated with this estimate is unknown but likely substantial, as assuming that all global mangrove systems emit at the same rate is unrealistic. However, we believe that it provides a first-order estimate of the potential GHG emissions from mangrove tree stems. We further compared the upscaled fluxes with the most recent estimates of ecosystem CH_4_ and N_2_O emissions from global mangroves, which combined eddy covariance (EC) measurements with various emission pathways, including stem-mediated emissions.^134^ The stem-mediated CH_4_ emissions estimated in this study were obviously lower than the ecosystem CH_4_ emissions (0.36 [0.23–0.54] Tg CH_4_ year^–1^) reported by Rosentreter et al.^134^ In contrast, the ecosystem N_2_O emissions (9.49 [4.67–20.69] Gg N_2_O year^–1^) were approximately 1000 times greater than the stem-mediated emissions in this study. We also converted the stem-mediated CH_4_ and N_2_O emissions into CO_2_e fluxes, resulting in total emissions of 2.52 Gg CO_2_e year^–1^. These emissions were further compared with the carbon burial rates of global mangroves, which average 72.82 Tg CO_2_e year^–1^.^135^ The offset from stem-mediated emissions was minimal. However, the contribution of tree fluxes to GHG emissions in mangroves has not reached a global consensus across studies, and research on this topic remains limited. Nevertheless, we postulate that this study offers extensive insight into GHG emissions from global mangroves by capturing the spatial and temporal variations in stem-mediated GHG emissions across multiple mangrove species.

**Table 4:** Upscaling Global Emissions of CH_4_ and N_2_O Mediated by Mangrove Stems

parameters of global mangroves	CH_4_	N_2_O
tree density (stem ha^–1^)^a^	263.68	
area (km^2^)^b^	145608	
stem-mediated emissions (Mg CH_4_/N_2_O year^–1^)^c^	5.94 [0.88–168.65]	8.66 [2.46–36.19]
stem-mediated emissions (Gg CO_2_e year^–1^)^d^	0.16 [0.02–4.55]	2.36 [0.67–9.88]
carbon burial rate (Tg CO_2_e year^–1^)^e^	72.82 [29.87–115.76]	
ecosystem emissions (Tg CH_4_/Gg N_2_O year^–1^)^f^	0.36 [0.23–0.54]	9.49 [4.67–20.69]

a The average tree density of global mangroves was obtained from Crowther et al.^132^

b The total area of global mangroves was obtained from Jia et al.^133^

c Stem-mediated CH_4_ and N_2_O emissions are presented as median [Q1–Q3] fluxes derived from this study.

d Stem-mediated CH_4_ and N_2_O emissions were converted to carbon dioxide (CO_2_)-equivalent fluxes using the global warming potential over a 100-year time scale (GWP_100_) in the IPCC AR6 report.

e The carbon burial rate of global mangroves was derived from Breithaupt and Steinmuller^135^ and is presented as the median [Q1–Q3]. This value was converted from carbon to CO_2_ using the ratio of the molar mass of CO_2_ to that of carbon. It was also recalculated using the aforementioned total area of global mangroves, which increased from 145,595 to 145,608 km^2^.

f Ecosystem emissions indicate the estimated CH_4_ and N_2_O emissions of global mangroves reported by Rosentreter et al., presented as the median [Q1–Q3].^134^ These values were recalculated using the aforementioned total area of global mangroves, which increased from 135,813 to 145,608 km^2^.

We acknowledge the limitations of the methods and materials used in this study, which may introduce some uncertainty in the results. First, the distinct design of the stem chamber in this study should be considered when discussing the differences in stem GHG flux among the species examined. One notable difference is the chamber’s transparency, which can lead to variations in stem GHG flux, as illustrated in Figure S4. Second, this study cannot confirm whether GHG emissions originate deep belowground from stem sources, primarily due to insufficient information about microbial communities and stable isotope signatures. Third, the carbon burial rate, which only considered autochthonous carbon inputs based on previous studies, was likely underestimated. This underestimation could lead to an overestimation of the offset value associated with stem-mediated CH_4_ and N_2_O emissions. Finally, global upscaling assumes uniform tree density and GHG flux rates across diverse biogeographic regions, which may overlook the known variability in mangrove structure and environmental drivers. Despite these limitations, this study provides valuable insights into the current knowledge gap regarding GHG emissions mediated by mangrove stems. These limitations also point to directions for future research. Specifically, there is a need to investigate soil microbial communities alongside flux measurements, standardize methods for measuring stem GHG flux, reassess carbon burial rates using long-term comprehensive data, and develop a refined global model that accounts for regional variations.

## Supplementary Material



## Data Availability

The original contributions presented in the study are included in the article. We encourage prospective data users to contact us before embarking on any analysis.

## Notes

Additional experimental methods, materials, and results include images of the sampling site and chamber design, variations in greenhouse gas fluxes, and comparisons of statistical models (PDF)
