# Supplementary material for: Exploring Spatial and Temporal Variations in Stem-Mediated Greenhouse Gas Emissions from Different Species of Mangroves
Source: Environ Sci Technol. 2025 Dec 3;59(49):26550–67. doi: 10.1021/acs.est.5c11290 (PMC12713777; doi:10.1021/acs.est.5c11290)
Supplement: Supplementary file 1 [file es5c11290_si_001.pdf]

Supporting information

Exploring spatial and temporal variations in stem-mediated greenhouse gas emissions from different species of mangroves

Zhao-Jun Yong[1], Wei-Jen Lin[2], Tzu-Chieh Chiu[3], Chen-Ying Ko[3], Pei-Luen Lu[4], Krisanadej Jaroensutasinee[5], Mullica Jaroensutasinee[5], Chiao-Wen Lin[3*], Hsing-Juh Lin[1*]

[1] Department of Life Sciences and Innovation and Development Center of Sustainable Agriculture, National Chung Hsing University, Taichung 402202, Taiwan

[2] Department of Biological Resources, National Chiayi University, Chiayi 600355, Taiwan

[3] Department of Marine Environment and Engineering, National Sun Yat-sen University, Kaohsiung 804201, Taiwan

[4] Department of Life Science, National Taitung University, Taitung 950309, Taiwan

[5] Center of Excellence for Ecoinformatics, School of Science, Walailak University, Nakhon Si Thammarat, Thailand

*Corresponding author: cwlin@g-mail.nsysu.edu.tw, hjlin@dragon.nchu.edu.tw

Summary of Contents:

- **Total Pages:** 16
- **Total Figures:** 5
- **Total Tables:** 3

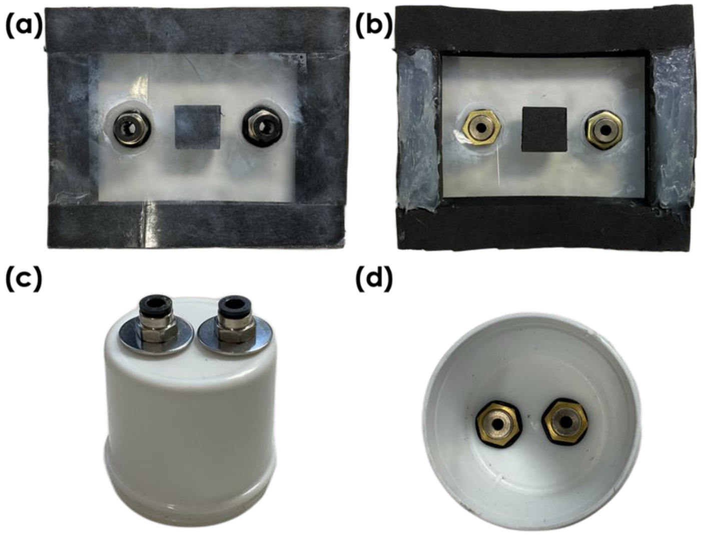

Figure S1. The design of the (a) semirigid chamber for *Kandelia obovata*, *Lumnitzera racemosa*, and *Rhizophora stylosa*, and (b) cylindrical chamber for *Avicennia marina*. The semirigid chamber was modified from Siegenthaler et al. (2016).

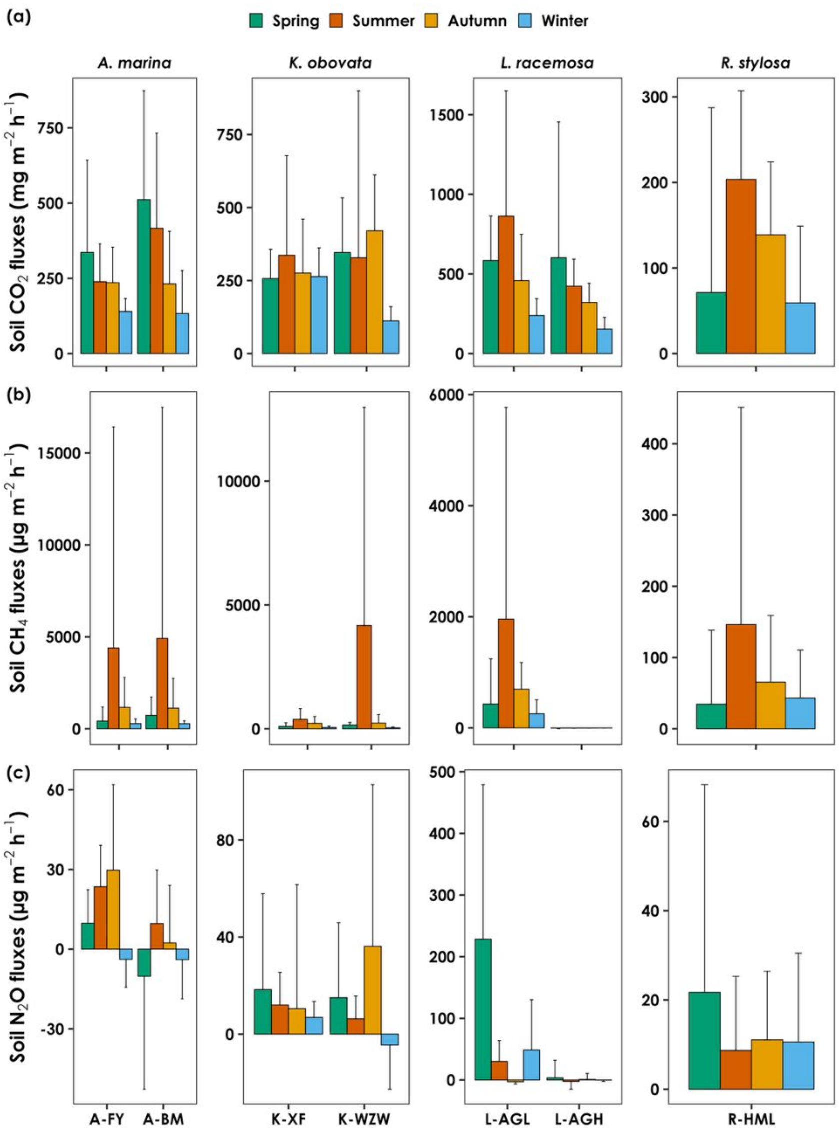

Figure S2. Seasonal variations of soil (a) carbon dioxide (CO_2), (b) methane (CH_4), and (c) nitrous oxide (N_2O) fluxes for each studied species. Note that the scale of the x-axis is different at each sub-plot.

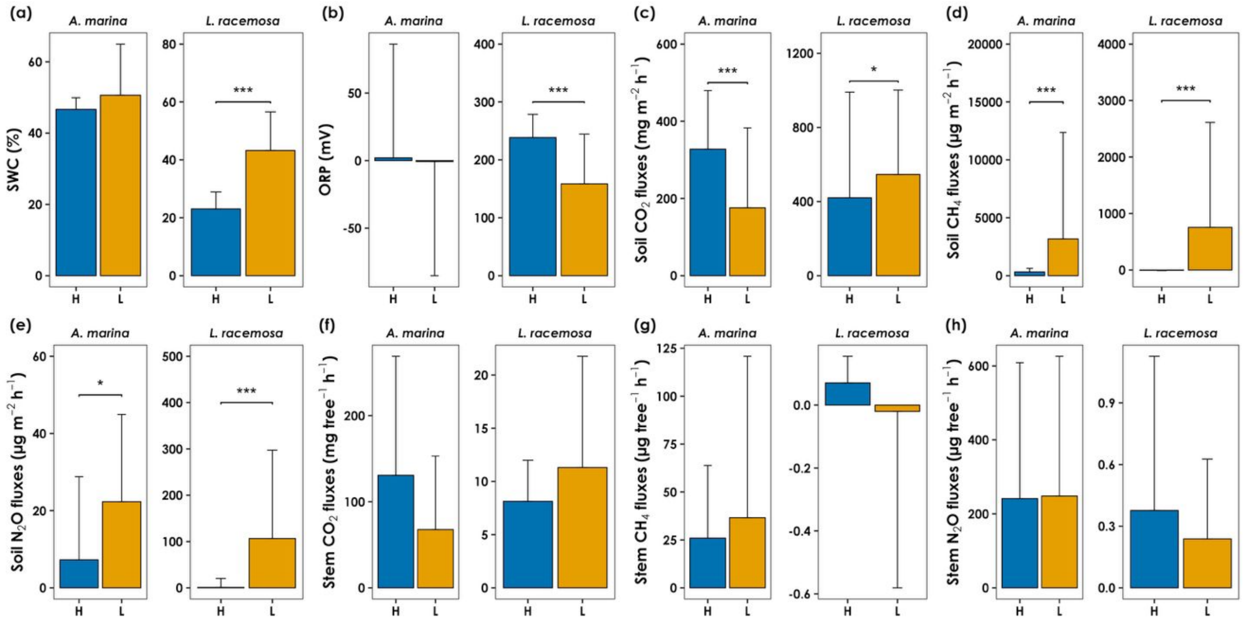

Figure S3. Comparison of soil conditions, stem and soil greenhouse gas (GHG) fluxes in *Avicennia marina* and *Lumnitzera racemosa* between sites with varying inundation durations: longer (H) and shorter (L). The annotations above the bar charts indicate significant differences among the sites.

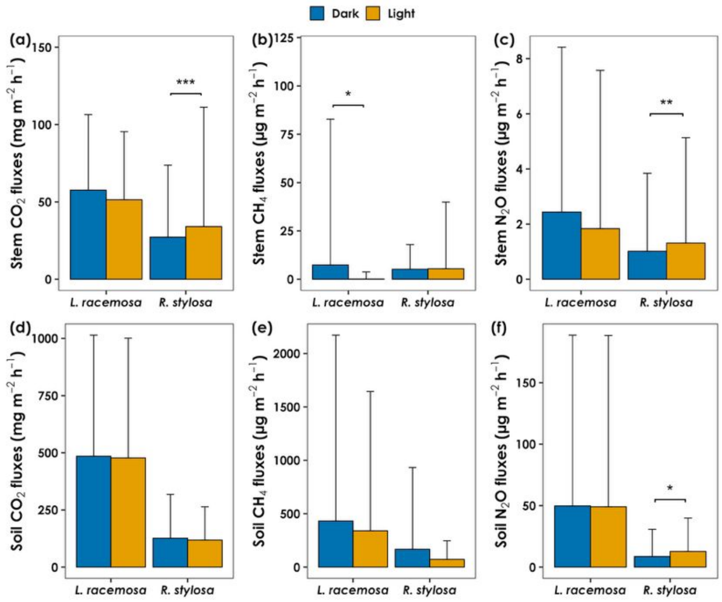

x

Figure S4. Comparison of stem and soil greenhouse gas (GHG) fluxes in *Lumnitzera racemosa* and *Rhizophora stylosa* measured under light and dark conditions. The dark condition was created by applying an opaque cloth to the stem chamber during GHG measurements to block sunlight.

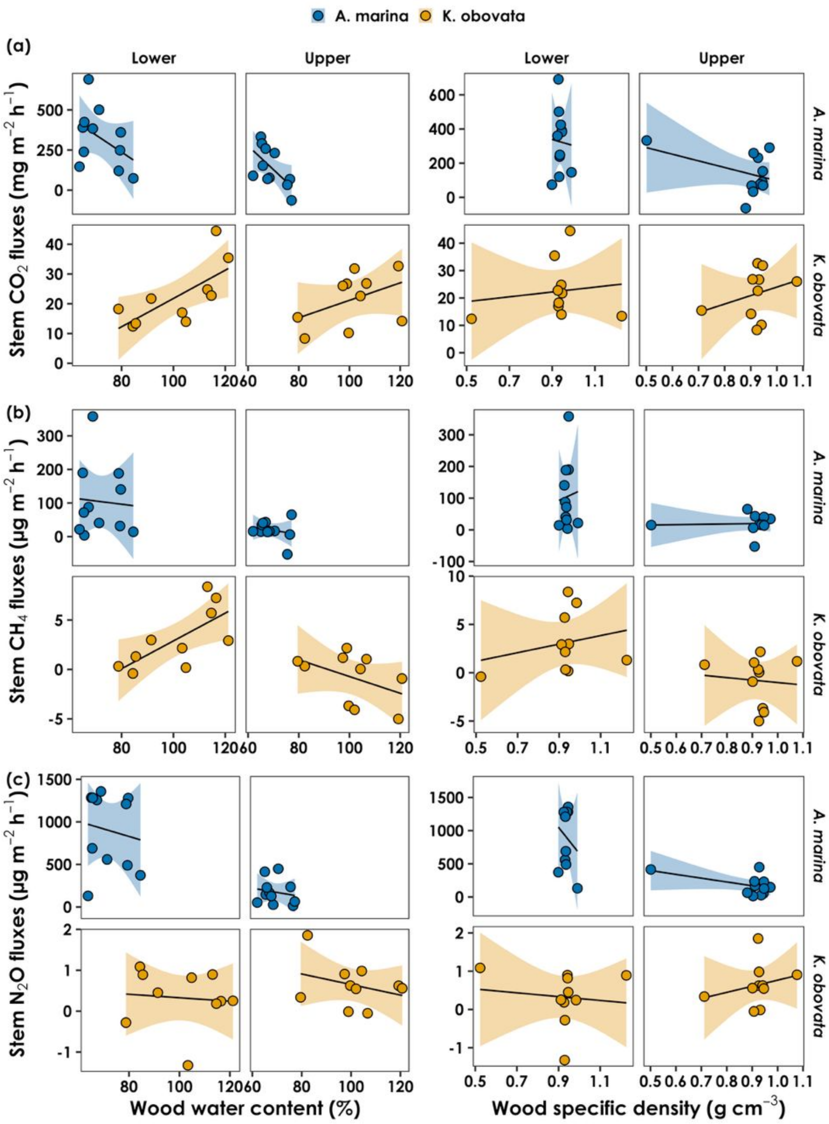

Figure S5. Correlations between stem (a) carbon dioxide (CO_2), (b) methane (CH_4), and (c) nitrous oxide (N_2O) flux and tree physiological factors. The results were divided into lower and upper parts of the stem based on height intervals. The range from 0 to 85 cm above ground was classified as lower, while the range from 85 to 200 cm above ground was classified as upper.

Table S1. Stem greenhouse gas (GHG) flux and tree physiological factors at different height intervals above the ground along the stem at all sites.

Site	Height interval (cm)	Stem CO_2 flux (mg m^{-2} h^{-1})	Stem CH_4 flux (μg m^{-2} h^{-1})	Stem N_2O flux (mg m^{-2} h^{-1})	Perimeter (m)	Lenticel density (m^{-2})	Wood specific density (g cm^{-3})	Wood water content (%)
L-AGH	0–30	28.32 ± 36.81^{a}	0.00 ± 0.83^{a}	0.56 ± 3.30^{a}	0.19 ± 0.03^{a}	1.21 ± 0.17^{a}	ND	ND
	30–60	53.12 ± 29.82^{b}	0.72 ± 1.42^{ab}	1.97 ± 5.45^{a}	0.15 ± 0.02^{b}	0.98 ± 0.43^{a}		
	60–90	57.52 ± 37.16^{b}	0.58 ± 0.59^{b}	2.41 ± 5.88^{a}	0.14 ± 0.02^{c}	0.79 ± 0.47^{ab}		
	90–120	44.55 ± 26.17^{ab}	0.42 ± 0.64^{ab}	3.73 ± 9.01^{a}	0.13 ± 0.01^{c}	0.53 ± 0.39^{b}		
L-AGL	0–30	71.06 ± 61.31^{a}	0.20 ± 3.28^{a}	0.65 ± 1.84^{a}	0.19 ± 0.05^{a}	1.13 ± 0.22^{a}	ND	ND
	30–60	65.39 ± 57.11^{a}	-1.05 ± 7.94^{a}	2.73 ± 7.44^{a}	0.15 ± 0.03^{b}	0.87 ± 0.41^{a}		
	60–90	49.43 ± 41.87^{a}	-0.81 ± 5.65^{a}	0.18 ± 3.21^{a}	0.13 ± 0.02^{bc}	0. ± 0.30^{b}		
	90–120	46.36 ± 47.84^{a}	1.04 ± 1.98^{a}	2.18 ± 5.55^{a}	0.12 ± 0.02^{c}	0.35 ± 0.22^{b}		
A-BM	0–40	586.23 ± 439.00^{a}	242.91 ± 630.63^{a}	1663.26 ± 1389.26^{a}	0.69 ± 0.1^{a}	1.75 ± 0.61^{a}	0.94 ± 0.01^{a}	67.61 ± 2.56^{a}
	40–85	369.85 ± 294.72^{ab}	28.91 ± 124.03^{ab}	633.01 ± 1029.37^{ab}	0.62 ± 0.13^{a}	2.55 ± 0.95^{c}		
	85–140	253.00 ± 231.68^{b}	32.96 ± 117.06^{b}	351.03 ± 680.84^{b}	0.49 ± 0.08^{c}	3.91 ± 1.47^{b}	0.85 ± 0.20^{a}	66.75 ± 2.21^{a}
	140–200	253.64 ± 257.93^{b}	27.21 ± 58.30^{b}	211.39 ± 271.39^{b}	0.41 ± 0.03^{b}	3.62 ± 0.57^{b}		
A-FY	0–40	249.06 ± 322.28^{a}	85.33 ± 189.86^{a}	762.89 ± 1190.78^{a}	0.47 ± 0.09^{a}	2.32 ± 1.43^{a}	0.94 ± 0.03^{a}	75.27 ± 8.55^{a}
	40–85	146.75 ± 196.03^{ab}	70.73 ± 228.65^{ab}	602.66 ± 959.02^{a}	0.41 ± 0.09^{ac}	1.94 ± 1.03^{a}		
	85–140	49.01 ± 153.18^{c}	26.19 ± 198.16^{b}	101.80 ± 281.60^{ab}	0.35 ± 0.08^{bc}	1.29 ± 0.68^{b}	0.92 ± 0.03^{a}	71.21 ± 6.12^{a}
	140–200	42.42 ± 186.95^{bc}	-4.70 ± 77.77^{b}	67.86 ± 155.14^{b}	0.32 ± 0.07^{b}	1.78 ± 0.73^{a}		
R-HML	0–40	35.92 ± 44.00^{a}	8.93 ± 48.65^{a}	1.09 ± 4.50^{a}	0.11 ± 0.02^{a}	0.12 ± 0.07^{a}	ND	ND
	40–80	32.36 ± 100.37^{a}	1.96 ± 3.55^{a}	1.54 ± 3.03^{a}	0.09 ± 0.02^{b}	0.12 ± 0.07^{a}		
K-WZW	0–40	23.64 ± 47.74^{a}	7.75 ± 15.90^{a}	0.43 ± 1.16^{a}	0.42 ± 0.04^{a}	0.06 ± 0.03^{a}	0.94 ± 0.03^{a}	111.37 ± 11.55^{a}
	40–85	36.04 ± 35.16^{a}	3.14 ± 8.96^{ab}	0.39 ± 1.25^{a}	0.38 ± 0.05^{b}	0.08 ± 0.04^{a}		
	85–140	19.41 ± 53.20^{a}	-4.05 ± 13.50^{b}	0.66 ± 1.46^{a}	0.36 ± 0.04^{b}	0.09 ± 0.04^{a}	0.93 ± 0.02^{a}	109.21 ± 10.01^{a}
	140–200	25.18 ± 31.64^{a}	-1.41 ± 10.06^{ab}	0.67 ± 1.81^{a}	0.36 ± 0.04^{b}	0.08 ± 0.04^{a}		
K-XF	0–40	12.55 ± 25.09^{a}	0.85 ± 4.15^{a}	0.51 ± 2.46^{a}	0.32 ± 0.16^{a}	0.03 ± 0.02^{a}	0.91 ± 0.25^{a}	91.35 ± 11.91^{a}
	40–85	17.45 ± 20.58^{a}	0.57 ± 3.91^{a}	-0.04 ± 3.71^{a}	0.28 ± 0.15^{b}	0.05 ± 0.03^{b}		

Site	Height interval (cm)	Stem CO_2 flux (mg m^{-2} h^{-1})	Stem CH_4 flux (μg m^{-2} h^{-1})	Stem N_2O flux (mg m^{-2} h^{-1})	Perimeter (m)	Lenticel density (m^{-2})	Wood specific density (g cm^{-3})	Wood water content (%)
	85–140	19.78 ± 29.69^{a}	1.06 ± 1.55^{a}	0.89 ± 3.15^{a}	0.25 ± 0.10^{b}	0.06 ± 0.03^{b}	0.91 ± 0.13^{a}	92.99 ± 11.58^{a}
	140–200	21.54 ± 16.32^{a}	1.16 ± 3.47^{a}	0.32 ± 1.32^{a}	0.24 ± 0.08^{b}	0.06 ± 0.04^{b}		

Table S2. Comparison of the RMSE values between the partial least squares structural equation modeling (PLS-SEM) and linear regression model (LM) benchmark.

Species	Model	Stem CO_2 flux	Stem CH_4 flux	Stem N_2O flux
Avicennia marina	PLS-SEM	162.9318	0.1607	0.3866
	LM	209.1087	0.1714	0.4257
Kandelia obovata	PLS-SEM	26.8788	0.0037	0.0008
	LM	30.1891	0.0036	0.0010
Lumitzera racemosa	PLS-SEM	37.3086	0.0023	0.0035
	LM	88.9478	0.0026	0.0042
Rhizophora stylosa	PLS-SEM	66.2931	0.0306	0.0030
	LM	182.6201	0.0336	0.0035

Stem CO_2, CH_4, and N_2O flux were the indicators for the construct TCO2, TCH4, and TN2O, respectively.

Table S3. The information related to the references corresponding to the IDs in Figure 6.

ID	Ecosystem	Period	Species	Height (m)	Technique	Stem CH_4 flux (μg m^{-2} h^{-1})	Stem N_2O flux (μg m^{-2} h^{-1})	Reference
A	Mangroves	Jul 2021 to Dec 2024	*Avecennia marina*	0–40	CEAS	-733.4 to 3381.45 (156.96 ± 451.22)	-26.03 to 4157.82 (1181.66 ± 1349.94)	This study
B	Mangroves	Jul 2021 to Dec 2024	*Avecennia marina*	40–85	CEAS	-521.35 to 1393.98 (51.72 ± 188.48)	-5.24 to 4162.28 (616.77 ± 980.48)	This study
C	Mangroves	Jul 2021 to Dec 2024	*Avecennia marina*	85–140	CEAS	-552.55 to 1137.82 (29.27 ± 165.3)	-234.49 to 2758.4 (220.48 ± 520.86)	This study
D	Mangroves	Jul 2021 to Dec 2024	*Avecennia marina*	140–200	CEAS	-393.72 to 261.24 (9.8 ± 70.99)	-19.41 to 948.69 (133.1 ± 225.04)	This study
E	Mangroves	Jul 2021 to Dec 2024	*Kandelia obovata*	0–40	CEAS	-17.14 to 61.48 (4.3 ± 12.04)	-6.83 to 6 (0.47 ± 1.9)	This study
F	Mangroves	Jul 2021 to Dec 2024	*Kandelia obovata*	40–85	CEAS	-13.06 to 41.08 (1.85 ± 6.98)	-14.65 to 4.46 (0.17 ± 2.74)	This study
G	Mangroves	Jul 2021 to Dec 2024	*Kandelia obovata*	85–140	CEAS	-67.41 to 8.42 (-1.5 ± 9.87)	-3.19 to 14.11 (0.78 ± 2.42)	This study
H	Mangroves	Jul 2021 to Dec 2024	*Kandelia obovata*	140–200	CEAS	-41.55 to 16.51 (-0.12 ± 7.57)	-1.94 to 5.76 (0.49 ± 1.57)	This study
I	Mangroves	Jul 2021 to Dec 2024	*Lumnitzera racemosa*	0–30	CEAS	-11.34 to 4.74 (0.09 ± 2.27)	-12.2 to 7.35 (0.6 ± 2.71)	This study
J	Mangroves	Jul 2021 to Dec 2024	*Lumnitzera racemosa*	30–60	CEAS	-31.24 to 18.04 (-0.08 ± 5.47)	-6.36 to 30.59 (2.32 ± 6.38)	This study
K	Mangroves	Jul 2021 to Dec 2024	*Lumnitzera racemosa*	60–90	CEAS	-19.46 to 2.39 (-0.05 ± 3.86)	-8.13 to 23.8 (1.4 ± 4.94)	This study
L	Mangroves	Jul 2021 to Dec 2024	*Lumnitzera racemosa*	90–120	CEAS	-2.01 to 8.52 (0.7 ± 1.44)	-11.31 to 38.52 (3.02 ± 7.61)	This study
M	Mangroves	Jul 2021 to Dec 2024	*Rhizophora stylosa*	0–40	CEAS	-6.32 to 337.47 (8.93 ± 48.65)	-19.68 to 8.94 (1.09 ± 4.5)	This study
N	Mangroves	Jul 2021 to Dec 2024	*Rhizophora stylosa*	40–80	CEAS	-5.00 to 13.15 (1.96 ± 3.55)	-7.18 to 10.24 (1.54 ± 3.03)	This study
1	Mangroves	Aug 2018	*Avecennia marina*	0.12	CRDS	0.2 to 336.91 (64.69 ± 18.51)	ND	Jeffrey et al. (2019)

ID	Ecosystem	Period	Species	Height (m)	Technique	Stem CH_4 flux (μg m^{-2} h^{-1})	Stem N_2O flux (μg m^{-2} h^{-1})	Reference
2	Mangroves	Aug 2018	*Avecennia marina*	0.4	CRDS	0.47 to 109.74 (19.38 ± 4.75)	ND	Jeffrey et al. (2019)
3	Mangroves	Aug 2018	*Avecennia marina*	0.8	CRDS	4.95 to 76.46 (20.12 ± 3.07)	ND	Jeffrey et al. (2019)
4	Mangroves	Feb 2012 to Nov 2013	*Laguncularia racemosa*	0.8	GC	(14.00 ± 13.00)	ND	He et al. (2019)
5	Mangroves	Feb 2012 to Nov 2013	*Sonneratia apetala*	0.8	GC	(42.00 ± 20.00)	ND	He et al. (2019)
6	Mangroves	Feb 2012 to Nov 2013	*Kandelia candel*	0.8	GC	(-29.00 ± 16.00)	ND	He et al. (2019)
7	Mangroves	Feb 2012 to Nov 2013	*Bruguiera gymnorhiza Bruguiera sexangula*	0.8	GC	(-7.90 ± 12.00)	ND	He et al. (2019)
8	Mangroves	Jul 2019 to Aug 2019	*Kandelia obovata (Site 1)*	0.4	CRDS	-1263.63 to 182.05 (-114.2)	ND	Gao et al. (2021)
9	Mangroves	Jul 2019 to Aug 2019	*Kandelia obovata (Site 1)*	1.4	CRDS	-844.83 to 142.6 (-70.42)	ND	Gao et al. (2021)
10	Mangroves	Jul 2019 to Aug 2019	*Kandelia obovata (Site 2)*	0.4	CRDS	-519.05 to 431.480 (47.64)	ND	Gao et al. (2021)
11	Mangroves	Jul 2019 to Aug 2019	*Kandelia obovata (Site 2)*	1.4	CRDS	-159.6 to 824.14 (26.15)	ND	Gao et al. (2021)
12	Mangroves	Jul 2019 to Aug 2019	*Avecennia marina*	0.4	CRDS	-544.72 to 360.90	ND	Gao et al. (2021)
13	Mangroves	Jul 2019 to Aug 2019	*Avecennia marina*	1.4	CRDS	-374.37 to 783.23	ND	Gao et al. (2021)
14	Mangroves	Jul 2019 to Aug 2019	*Aegiceras corniculatum*	0.4	CRDS	-2104.29 to 3611.57	ND	Gao et al. (2021)
15	Mangroves	Jul 2019 to Aug 2019	*Aegiceras corniculatum*	1.4	CRDS	-664.38 to 680.58	ND	Gao et al. (2021)
16	Mangroves	Jan, Jul 2018	*Kandelia obovata*	0 to 1.25	GC	(112.95 ± 63.49)	ND	Zhang et al. (2022)

ID	Ecosystem	Period	Species	Height (m)	Technique	Stem CH_4 flux (μg m^{-2} h^{-1})	Stem N_2O flux (μg m^{-2} h^{-1})	Reference
17	Mangroves	Jan, Jul 2018	*Aegiceras corniculatum*	0 to 1.25	GC	(86.88 ± 48.79)	ND	Zhang et al. (2022)
18	Mangroves	Jan, Jul 2018	*Avecennia marina*	0 to 1.25	GC	(44.78 ± 34.08)	ND	Zhang et al. (2022)
19	Mangroves	Dec 2021 to Mar 2022	*Kandelia obovata*	0.7 to 1.7	GC	-44.13 to 88.00 (8.71 ± 1.53)	-9.49 to 28.35 (2.09 ± 0.21)	Liao et al. (2024)
20	Mangroves	Dec 2021 to Mar 2022	*Senneratia apetala*	0.7 to 1.7	GC	-26.67 to 97.33 (13.32 ± 1.48)	-6.73 to 28.95 (2.05 ± 0.19)	Liao et al. (2024)
21	Mangroves	Jul 2022	*Bruguiera gymnorhiza*	0.3	CEAS	28.87 to 13234.92 (2303.99)	ND	Epron et al. (2023)
22	Mangroves	Jul 2022	*Bruguiera gymnorhiza*	0.3 to 2.0	CEAS	-5.77 to 4221.09 (490.82)	ND	Epron et al. (2023)
23	Mangroves	Jun 2022 to Jul 2022	*Kandelia obovata*	1.1	CEAS	-0.80 to 2.89 (0.64 ± 0.64)	ND	Yong et al. (2024)
24	Mangroves	Jun 2022 to Jul 2022	*Kandelia obovata*	1.1	CEAS	-30.80 to 8.82 (-2.73 ± 8.34)	ND	Yong et al. (2024)
25	Mangroves	Jun 2022 to Jul 2022	*Avicennia marina*	1.1	CEAS	-66.25 to 42.83 (7.70 ± 18.77)	ND	Yong et al. (2024)
26	Mangroves	Jun 2022 to Jul 2022	*Avicennia marina*	1.1	CEAS	-4.13 to 2.67 (0.48 ± 1.17)	ND	Yong et al. (2024)
27	Floodplain	Jul 2021 to Nov 2013	*Fraxinus mandshurica*	0.15	GC	81 to 1514	ND	Terazawa et al. (2015)
28	Peatlands	Oct 2020 to May 2021	*Betula pubescens*	0.1 to 1.7	GC	(0.24 ± 0.04)	(4.52 ± 0.06)	Ranniku et al. (2023)
29	Peatlands	Oct 2020 to May 2021	*Picea abies*	0.1 to 1.7	GC	(0.13 ± 0.04)	(0.01 ± 0.03)	Ranniku et al. (2023)
30	Riparian	May to Sep 2017	*Populus angustifolia, Populus deltoides*	0.65	CEAS	(0.06 ± 0.08)	ND	Flanagan et al. (2021)
31	Riparian	Sep 2017 to Dec 2018	*Alnus incana*	0.1 to 1.7	GC	ND	-125.69 to 4713.42 (0.69 ± 0.22)	Mander et al. (2021)
32	Floodplain	May to Oct 2005	*Fraxinus mandshurica*	0.15	GC	(176)	ND	Terazawa et al. (2007)

ID	Ecosystem	Period	Species	Height (m)	Technique	Stem CH_4 flux (µg m^{-2} h^{-1})	Stem N_2O flux (µg m^{-2} h^{-1})	Reference
33	Floodplain	May to Oct 2005	*Fraxinus mandshurica*	0.7	GC	(97)	ND	Terazawa et al. (2007)
34	Tropical wetlands	Oct 2017	ND	0.2–1.1	CEAS	(0.09 ± 0.03)	(0.26 ± 0.46)	Cugler et al. (2024)
35	Tropical wetlands	Oct 2017	ND	0.2–1.1	CEAS	(0.15 ± 0.04)	(0.20 ± 0.07)	Cugler et al. (2024)
36	Peatlands	Sep 2019 to Mar 2020	*Symphonia globulifera*	0.3	CEAS	(8.96 ± 2.04)	(0.09 ± 0.16)	Cugler et al. (2024)
37	Peatlands	Sep 2019 to Mar 2020	*Symphonia globulifera*	0.8	CEAS	(6.84 ± 1.7)	(0.09 ± 0.03)	Cugler et al. (2024)
38	Peatlands	Sep 2019 to Mar 2020	*Symphonia globulifera*	1.7	CEAS	(6.2 ± 1.22)	(-0.16 ± 0.09)	Cugler et al. (2024)
39	Peatlands	Sep 2019 to Mar 2020	*Mauritia flexuosa*	0.3	CEAS	(1056.42 ± 140.23)	(-0.13 ± 0.09)	Cugler et al. (2024)
40	Peatlands	Sep 2019 to Mar 2020	*Mauritia flexuosa*	0.8	CEAS	(2138.22 ± 221.57)	(0.28 ± 0.25)	Cugler et al. (2024)
41	Peatlands	Sep 2019 to Mar 2020	*Mauritia flexuosa*	1.7	CEAS	(1061.77 ± 91.08)	(-0.03 ± 0.22)	Cugler et al. (2024)
42	Boreal forests	Jun to May 2015	*Pinus sylvestris*	0.2	GC	ND	(0.1)	Machacova et al. (2019)
43	Boreal forests	Jun to May 2015	*Picea abies*	0.2	GC	ND	(0.05)	Machacova et al. (2019)
44	Boreal forests	Jun to May 2015	*Betula pubescens, B. pendula*	0.2	GC	ND	(0.04)	Machacova et al. (2019)
45	Tropical rainforests	Oct to Nov 2018	*Syzygium borbonicum, Doratoxylon apetalum, Antirhea borbonica, Homalium paniculatum, Mimusops balata, Labourdonnaisia calophylloides*	0.4, 1.1, 1.8	FTIR	(-15.60 ± 2.00)	(-3.00 ± 0.80)	Machacova et al. (2021)

ID	Ecosystem	Period	Species	Height (m)	Technique	Stem CH_4 flux (μg m^{-2} h^{-1})	Stem N_2O flux (μg m^{-2} h^{-1})	Reference
46	Mesocosms	Jun 2009 to Jul 2010	*Alnus glutinosa*	0.1	GC	0.96 to 56.50	7.88 to 5846.00	Machacova et al. (2013)
47	Mesocosms	Jun 2009 to Jul 2010	*Fagus sylvatica*	0.1	GC	-2.25 to 1.93	0.32 to 4555.00	Machacova et al. (2013)
48	Tropical forests	Mar to Jul 2014	*Simarouba amara*	0.3	OA-ICOS	-276 to 678 (87.7 ± 18.5)	-3770 to 8361 (1193 ± 361)	Welch et al. (2019)
49	Tropical forests	Mar to Jul 2014	*Heisteria concinna*	0.3	OA-ICOS	-156 to 598 (101 ± 14.9)	-2857 to 4270 (80 ± 234)	Welch et al. (2019)
50	Hemi-boreal forests	Jul 2017 to Sep 2018	*Alnus incana*	0.1	CRDS	-0.53 to 225.00	0.13 to 46.91	Schindler et al. (2021)
51	Hemi-boreal forests	Jul to Sep 2017	*Alnus incana*	0.1	GC	ND	ND	Schindler et al. (2020)
52	Temperate forests	Jun 2019	*Fagus sylvatica*	0.9, 1.8	FTIR	ND	(-0.254 ± 0.827)	Machacova et al. (2024)
53	Tropical forests	Oct to June 2019	*Eperua falcata*	1.3	FTIR	-5.11 to 15.24 (1.96 ± 1.84)	-13.68 to 0.46 (-0.96 ± 1.47)	Brechet et al. (2025)
54	Tropical forests	Oct to June 2019	*Lecythis poiteaui*	1.3	FTIR	-0.97 to 38.95 (5.26 ± 3.41)	-4.46 to 4.96 (-0.48 to 0.60)	Brechet et al. (2025)
55	Temperate forests	Nov 2017 to Dec 2018	*Fagus sylvatica*	0.4	GC	-4.37 to 173.97	ND	Machacova et al. (2023)
56	Temperate forests	Apr to Oct 2016	*Quercus petraea*	0.25	OA-ICOS	(0.52 ± 0.35)	ND	Plain et al. (2019)
57	Subtropical forests	Jan to Dec 2020	*Populus deltoides* × *P. euramericana*	0.5, 1.0, 1.5	GC	20.83 to 512.5 (112.5 ± 16.67)	ND	Feng et al. (2022)
58	Boreal forests	May to Jul 2013	*Pinus sylvestris*	0.2	GC	Median: 0.005	Median: 0.023	Machacova et al. (2016)
59	Hemi-boreal forests	Sep 2017 to Dec 2018	*Alnus incana*	0.1, 0.8, 1.7	GC	ND	0.88 to 71.64 (0.69 ± 0.22)	Mander et al. (2021)
60	Tropical rainforests	May 2017 to Apr 2018	*Allanblackia floribunda,*	1.3	GC	ND	(3.55 ± 0.69)	Iddris et al. (2020)

ID	Ecosystem	Period	Species	Height (m)	Technique	Stem CH_4 flux (μg m^{-2} h^{-1})	Stem N_2O flux (μg m^{-2} h^{-1})	Reference
			Anthonotha macrophylla, Cleistopholis patens, Coelocaryon preussi, Desbordesia insignis, Parkia bicolor, Plagiostyles africana, Pycnanthus angolensis, Staudtia kamerunensis, Theobroma cacao					
61	Tropical rainforests	May 2017 to Apr 2018	*Carapa procera, Celtis* sp., *Diospyros* sp., *Entandrophragma candollei, Eribroma oblongum, Lovoa trichilioides, Petersianthus macrocarpus, Theobroma cacao*	1.3	GC	ND	(7.48 ± 1.51)	Iddris et al. (2020)
62	Tropical rainforests	May 2017 to Apr 2018	*Annickia chlorantha, Anonidium mannii, Carapa procera, Celtis* sp., *Funtumia elastica, Leonardoxa africana, Markhamia lutea, Mitragyna stipulosa,*	1.3	GC	ND	(2.80 ± 0.31)	Iddris et al. (2020)

ID	Ecosystem	Period	Species	Height (m)	Technique	Stem CH_4 flux (µg m^{-2} h^{-1})	Stem N_2O flux (µg m^{-2} h^{-1})	Reference
			Pycnanthus angolensis, Theobroma cacao					

GC: Gas chromatography; OA-ICOS: Off-axis integrated cavity output spectroscopy; CEAS: Cavity-enhanced absorption spectroscopy; FTIR: Fourier transform infrared spectroscopy

Reference

Bonal, D.; Janssens, I. A. Mangrove Production and Carbon Sinks: A Revision of Global Budget Estimates. *New Phytol.* **2025**, 2451–2466.

Cugler, G.; Figueiredo, V.; Gauci, V.; Stauffer, T.; Bittencourt Peixoto, R.; Rao Pangala, S.; Enrich-Prast, A. Analysis of CH_4 and N_2O Fluxes in the Dry Season: Influence of Soils and Vegetation Types in the Pantanal. *Forests* **2024**, *15* (12), 2224.

Feng, H.; Guo, J.; Ma, X.; et al. Methane Emissions May Be Driven by Hydrogenotrophic Methanogens Inhabiting the Stem Tissues of Poplar. *New Phytologist* **2022a**, *233* (1), 182–193. https://doi.org/10.1111/nph.17778.

Flanagan, L. B.; Nikkel, D. J.; Scherloski, L. M.; Tkach, R. E.; Smits, K. M.; Selinger, L. B.; Rood, S. B. Multiple Processes Contribute to Methane Emission in a Riparian Cottonwood Forest Ecosystem. *New Phytol.* **2021**, *229*, 1970–1982.

Gao, C. H.; Zhang, S.; Ding, Q. S.; Wei, M. Y.; Li, H.; Li, J.; Wen, C.; Gao, G. F.; Liu, Y.; Zhou, J. J.; et al. Source or Sink? A Study on the Methane Flux From Mangroves Stems in Zhangjiang Estuary, Southeast Coast of China. *Sci. Total Environ.* **2021**, *788*, 147782.

He, Y.; Guan, W.; Xue, D.; Liu, L.; Peng, C.; Liao, B.; Hua, J.; Zhu, Q.; Yang, Y.; Wang, X.; Zhou, G.; Wu, Z.; Chen, H. Comparison of Methane Emissions among Invasive and Native Mangrove Species in Dongzhaigang, Hainan Island. *Sci. Total Environ.* **2019**, *697*, 133945.

Iddris, N. A.-A.; Corre, M. D.; Yemefack, M.; van Straaten, O.; Veldkamp, E. Stem and Soil Nitrous Oxide Fluxes from Rainforest and Cacao Agroforest on Highly Weathered Soils in the Congo Basin. *Biogeosciences* **2020**, *17*, 5377–5397.

Jeffrey, L. C.; Reithmaier, G.; Sippo, J. Z.; Johnston, S. G.; Tait, D. R.; Harada, Y.; Maher, D. T. Are Methane Emissions from Mangrove Stems a Cryptic Carbon Loss Pathway? Insights from a Catastrophic Forest Mortality. *New Phytol.* **2019**, *224* (1), 146–154.

Liao, X.; Wang, Y.; Malghani, S.; Zhu, X.; Cai, W.; Qin, Z.; Wang, F. Methane and Nitrous Oxide Emissions and Related Microbial Communities from Mangrove Stems on Qi'ao Island, Pearl River Estuary in China. *Sci. Total Environ.* **2024**, *915*, 170062.

Machacova, K.; Bäck, J.; Vanhatalo, A.; Halmeenmäki, E.; Kolari, P.; Mammarella, I.; Pumpanen, J.; Acosta, M.; Urban, O.; Pihlatie, M. *Pinus Sylvestris* as a Missing Source of Nitrous Oxide and Methane in Boreal Forest. *Sci. Rep.* **2016**, *6* (1), 23410.

Machacova, K.; Borak, L.; Agyei, T.; Schindler, T.; Soosaar, K.; Mander, Ü.; Ah-Peng, C. Trees as Net Sinks for Methane (CH_4) and Nitrous Oxide (N_2O) in the Lowland Tropical Rain Forest on Volcanic Réunion Island. *New Phytol.* **2021**, *229* (4), 1983–1994.

Machacova, K.; Papen, H.; Kreuzwieser, J.; Rennenberg, H. Inundation Strongly Stimulates Nitrous Oxide Emissions from Stems of the Upland Tree *Fagus Sylvatica* and the Riparian Tree *Alnus Glutinosa*. *Plant Soil* **2013**, *364*, 287–301. *https://doi.org/10.1007/s11104-012-1359-4*.

Machacova, K.; Schindler, T.; Bréchet, L.; Mander, Ü.; Grams, T. E. E. Substantial Uptake of Nitrous Oxide (N_2O) by Shoots of Mature European Beech. *Sci. Total Environ.* **2024**, *934*, 173122.

Machacova, K.; Vainio, E.; Urban, O.; Pihlatie, M. Seasonal Dynamics of Stem N_2O Exchange Follow the Physiological Activity of Boreal Trees. *Nat. Commun.* **2019**, *10*, 4989.

Machacova, K.; Warlo, H.; Svobodová, K.; Agyei, T.; Uchytilová, T.; Horáček, P.; Lang, F. Methane Emission from Stems of European Beech (*Fagus sylvatica*) Offsets as Much as Half of Methane Oxidation in Soil. *New Phytol.* **2023**, *238*, 584–597.

Mander, Ü.; Krasnova, A.; Escuer-Gatius, J.; Espenberg, M.; Schindler, T.; Machacova, K.; Pärn, J.; Maddison, M.; Megonigal, J. P.; Pihlatie, M.; Kasak, K.; Niinemets, Ü.; Junninen, H.; Soosaar, K. Forest Canopy Mitigates Soil N_2O Emission during Hot Moments. *npj Clim. Atmos. Sci.* **2021**, *4* (1), 1–9.

Plain, C.; Ndiaye, F.-K.; Bonnaud, P.; Ranger, J.; Epron, D. Impact of Vegetation on the Methane Budget of a Temperate Forest. *New Phytol.* **2019**, *221* (3), 1447–1456.

Ranniku, R.; Schindler, T.; Escuer-Gatius, J.; Mander, Ü.; Machacova, K.; Soosaar, K. Tree Stems Are a Net Source of CH_4 and N_2O in a Hemiboreal Drained Peatland Forest during the Winter Period. *Environ. Res. Commun.* **2023**, *5* (5), 051010.

Schindler, T.; Machacova, K.; Mander, Ü.; Escuer-Gatius, J.; Soosaar, K. Diurnal Tree Stem CH_4 and N_2O Flux Dynamics from a Riparian Alder Forest. *Forests* **2021**, *12* (7), 863.

Schindler, T.; Mander, Ü.; Machacova, K.; Espenberg, M.; Krasnov, D.; Escuer-Gatius, J.; Veber, G.; Pärn, J.; Soosaar, K. Short-Term Flooding Increases CH_4 and N_2O Emissions from Trees in a Riparian Forest Soil-Stem Continuum. *Sci. Rep.* **2020**, *10*, 3204.

Siegenthaler, A.; Welch, B.; Pangala, S. R.; Peacock, M.; Gauci, V. Technical Note: Semi-Rigid Chambers for Methane Gas Flux Measurements on Tree Stems. *Biogeosciences* **2016**, *13*, 1197–1207.

Terazawa, K.; Ishizuka, S.; Sakata, T.; Yamada, K.; Takahashi, M. Methane Emissions from Stems of *Fraxinus Mandshurica* Var. *Japonica* Trees in a Floodplain Forest. *Soil Biol. Biochem.* **2007**, *39* (10), 2689–2692.

Terazawa, K.; Yamada, K.; Ohno, Y.; Sakata, T.; Ishizuka, S. Spatial and Temporal Variability in Methane Emissions from Tree Stems of *Fraxinus Mandshurica* in a Cool-Temperate Floodplain Forest. *Biogeochemistry* **2015**, *123* (3), 349–362.

Welch, B.; Gauci, V.; Sayer, E. J. Tree Stem Bases Are Sources of CH_4 and N_2O in a Tropical Forest on Upland Soil during the Dry to Wet Season Transition. *Global Change Biol.* **2019**, *25*, 361–372.

Yong, Z.-J.; Lin, W.-J.; Lin, C.-W.; Lin, H.-J. Tidal Influence on Carbon Dioxide and Methane Fluxes from Tree Stems and Soils in Mangrove Forests. *Biogeosciences* **2024**, *21* (22), 5247–5260.

Zhang, C.; Zhang, Y.; Luo, M.; Tan, J.; Chen, X.; Tan, F.; Huang, J. Massive Methane Emission from Tree Stems and Pneumatophores in a Subtropical Mangrove Wetland. *Plant Soil* **2022**, *473* (1), 489–505.
